# Internal Promoters and Their Effects on the Transcription of Operon Genes for Epothilone Production in *Myxococcus xanthus*

**DOI:** 10.3389/fbioe.2021.758561

**Published:** 2021-10-27

**Authors:** Ye Wang, Xin-jing Yue, Shu-fei Yuan, Yu Hong, Wei-feng Hu, Yue-zhong Li

**Affiliations:** State Key Laboratory of Microbial Technology, Institute of Microbial Technology, Shandong University, Qingdao, China

**Keywords:** internal promoters, epothilone, biosynthetic gene cluster, operon, transcription, CRISPR-dCas9 activation, *Myxococcus xanthus*

## Abstract

The biosynthetic genes for secondary metabolites are often clustered into giant operons with no transcription terminator before the end. The long transcripts are frangible and the transcription efficiency declines along with the process. Internal promoters might occur in operons to coordinate the transcription of individual genes, but their effects on the transcription of operon genes and the yield of metabolites have been less investigated. Epothilones are a kind of antitumor polyketides synthesized by seven multifunctional enzymes encoded by a 56-kb operon. In this study, we identified multiple internal promoters in the epothilone operon. We performed CRISPR-dCas9–mediated transcription activation of internal promoters, combined activation of different promoters, and activation in different epothilone-producing *M. xanthus* strains. We found that activation of internal promoters in the operon was able to promote the gene transcription, but the activation efficiency was distinct from the activation of separate promoters. The transcription of genes in the operon was influenced by not only the starting promoter but also internal promoters of the operon; internal promoters affected the transcription of the following and neighboring upstream/downstream genes. Multiple interferences between internal promoters thus changed the transcriptional profile of operon genes and the production of epothilones. Better activation efficiency for the gene transcription and the epothilone production was obtained in the low epothilone-producing strains. Our results highlight that interactions between promoters in the operon are critical for the gene transcription and the metabolite production efficiency.

## Introduction

Operons are clusters of prokaryotic genes that are co-transcribed and functionally related (Osbourn and Field, 2009). Large operons, such as those involved in many pathways for the biosynthesis of secondary metabolites in prokaryotes, contain multiple genes with no transcription terminator before the end. The transcription of multiple genes in operons is initiated by the starting promoter and forms a single large polycistronic mRNA molecule. Large mRNAs are easily subject to various influences in cells, leading to not only frangibility of the mRNA molecules but also low efficiency of the transcriptional process. According to experiments and bioinformatic predictions, internal promoters might be universal in bacterial operons to coordinate the transcriptional process (Ma et al., 1981; Kaebernick et al., 2002; Makita et al., 2004; Güell et al., 2009; Jones et al., 2009; Sharma et al., 2010). For example, the *rpoBC* operon of *E. coli* encodes four ribosomal proteins and the β and β’ subunits of RNA polymerase, and this operon contains at least three weak internal promoters P2, P3, and P4 in addition to two strong promoters P_L11_ and P1 (Ma et al., 1981). In the 57-kb jamaicamide operon from the marine cyanobacterium *Lyngbya majuscula*, 17 genes are co-transcribed from the starting promoter and six internal promoters were found in the intergenic regions of the operon, which were suggested to manage the toxin production in various environments (Jone et al., 2009). Similarly, seven internal promoters were observed in the microcystin operon of *Microcystis aeruginosa* (Kaebernick et al., 2002). However, internal promoters, especially multiple internal promoters, have been less investigated for their effects on the transcriptional processes of operon genes and the yields of secondary metabolites.

Epothilones were originally discovered in the extracts of some *Sorangium cellulosum* strains and are a kind of antitumor polyketides with microtubule-stabilizing activity (Bollag et al., 1995; Gerth et al., 1996). The epothilone gene cluster is approximately 56 kb in size, containing seven large open reading frames (ORFs) in the same transcriptional direction (Julien et al., 2000; Molnár et al., 2000). These ORFs are predicted to encode eleven functional modules, including nine polyketide synthase modules by the *epoA*, *epoB*, *epoC*, *epoD*, and *epoE* ORFs; one nonribosomal peptide synthetase module by *epoP*; and one cytochrome P450 cyclooxygenase module by *epoF*. These enzymes process the elongation, modification, and release of epothilones in a sequential mode. The epothilone gene cluster contains no terminator between ORFs before the end and is thus suggested to form a giant operon.

*S. cellulosum* So0157-2 is an epothilone producer (Han et al., 2013), and the epothilone biosynthetic gene cluster from this strain has been successfully expressed in *Myxococcus xanthus* (Zhu et al., 2015). We found that transcription of the epothilone operon genes greatly varied in either *M. xanthus* or the original *S. cellulosum* producers. Although the production of epothilones in *M. xanthus* has been greatly improved by different engineering techniques (Zhu et al., 2015; Yue et al., 2017; Peng et al., 2018; Yue et al., 2018), the transcription levels of operon genes still varied. Uneven expression of operon genes has been observed for many years (Murakawa et al., 1991), and genome-wide transcriptomic studies have also revealed varied transcription levels of consecutive operon genes (de-Hoon et al., 2005). The uneven expressions imply that transcription of multiple operon genes is complexly regulated, rather than controlled only by the starting promoter and the operator. We suggested that internal promoters might play an important role in coordinating the transcription of operon genes for the production of epothilones.

Previously, we identified the starting promoter of the epothilone gene cluster from So0157-2 and found two transcription start sites, named TSS1 and TSS2, which are located 246 bp and 193 bp upstream of the translation start site of the epothilone operon (Zhu et al., 2013). In addition, there is a 24-bp stem-loop (hairpin) structure in the starting promoter, which plays a negative regulation role in the transcription (Zhu et al., 2013). The transcription regulator Esi was able to bind to the hairpin sequence to downregulate the transcriptional level of the epothilone biosynthetic gene cluster (Yue et al., 2017). In this study, we identified multiple internal promoters in the epothilone biosynthetic gene clusters. To determine their effects on the transcription, we employed the established CRISPRa (CRISPR-dCas9–mediated transcription activation) technique (Peng et al., 2018) to change the activities of internal promoters, combined the activation of different promoters, and performed activation in different epothilone-producing *M. xanthus* strains. We found that activation of the internal promoters was able to promote the transcriptional levels of operon genes, and thus the yields of epothilones. However, the activation efficiency in the operon was distinct from that in separate forms. Normally, internal promoters interfered with neighboring promoters to coordinate the transcriptional processes of operon genes and the production of epothilones. The potential regulation mechanisms of internal promoters in operons are discussed.

## Materials and Methods

### Strains and Culture Conditions

Strains used in this study are listed in Supplementary Table S1.

*Escherichia coli* DH5α and HB101 were used for routine transformation and subcloning. The *E. coli* strains were grown routinely in Luria Broth (LB) medium (10 g/L peptone, 5 g/L yeast extract, and 5 g/L NaCl, pH 7.2). *M. xanthus* strains were grown in CYE medium [10 g/L casitone, 5 g/L yeast extract, 10 mM 3-(N-morpholino) propanesulfonic acid (MOPS; pH 7.6), and 4 mM MgSO_4_] for routine growth and CMO medium (CYE medium plus 7 mL/L methyl oleate) for the production of epothilones. When appropriate, kanamycin (40 μg/mL), tetracycline (10 μg/mL), and apramycin (25 μg/mL) were added. The growth temperatures were 37°C for *E. coli* and 30°C for *M. xanthus*.

### Detection of Gene Co-Transcription

Total RNA was extracted from cell cultures using BIOZOL kits (Total RNA Extraction Reagent, BioFast, China) and then transcribed reversely into cDNA with PrimeScript™ reagent kit with DNAase (Takara, Japan). Primers were designed at the junction between two genes (Supplementary Table S2). Total RNA without inverse transcription and the cells containing the epothilone gene cluster were used as templates in the negative and positive control groups, respectively. In experimental groups, the cDNAs were used as templates.

### Prediction of Internal Promoters

We used the promoter prediction software “Neural Network Promoter Prediction” (https://www.fruitfly.org/seq_tools/promoter.html) to predict internal promoters in the epothilone gene cluster derived from *Sorangium cellulosum* strain So0157-2. The threshold was set as 0.8. At the same time, another online promoter prediction software “BPROM” (http://www.softberry.com/berry.phtml) was also employed in the prediction. The −35 and −10 binding regions were predicted in comparison to the σ70 consensus -35 (TTGACA) and -10 (TATAAT) promoter regions of *E. coli*. The threshold was set greater than 0.8.

### Construction of Plasmids

The primers and plasmids used in this study are provided in Supplementary Tables S2 and S3.

The plasmids pkk-232-P_epoP_, pkk-232-P_epoB_ ∼ pkk-232-P_epoF_ were used as a promoter activity reporter vector in *E. coli* (Supplementary Figure S1). The promoters *P*_*epoP*_, *P*_*epoB*_ ∼ *P*_*epoF*_ were obtained by PCR with primers P_epoP_-F/R, P_epoB_-F/R ∼ P_epoF_-F/R from the epothilone gene cluster in So0157-2. The plasmids pkk-232-P_epoP_, pkk-232-P_epoB_ ∼ pkk-232-P_epoF_ were constructed by inserting *P*_*epoP*_, *P*_*epoB*_ ∼ *P*_*epoF*_ into the HindⅢ/BamHⅠ sites of plasmid pkk-232-8. The above plasmids were used to detect the activity of internal promoters from the epothilone operon in *E. coli*. The plasmid pkk-232-aphⅡ was constructed by inserting the promoter *aphⅡ* into the HindⅢ/BamHⅠ sites of pkk-232-8 and employed as the positive control. The promoter activity was characterized by detecting the activity of the report gene chloramphenicol acetyltransferase (*CAT*). The activity of the reporter gene *CAT* was detected by using the CAT ELISA kit. The CAT ELISA kit was purchased from Roche and operated according to the instructions provided.

In our previous work (Peng et al., 2018), we constructed the plasmid pSWcuomxdCas9-ω (Supplementary Figure S2). It contains codon-optimized *mxdcas9* [D10A (GAC→GGC) and H840A (CAC→GCC)], linked to the EcoRI/HindⅢ site of the pSWU30 plasmid. We fused the *Omega* (*ω*) protein gene from *M. xanthus* DK1622 to the 3′-end of *mxdcas9* with a 21-nt linker (AAG​CTT​TCT​GGA​TCA​AGT​TCT). In front of *mxdcas9* is the promoter P_*cuoA*_ from *M. xanthus* DK1622, which is inducible by the addition of cupric ions (200 μM).

The plasmid pZJY41-sgRNA was used to express sgRNA (Supplementary Figure S3). To construct the pZJY41-sgRNA series plasmids, we designed spacers targeting different internal promoters with the online software “CasOT” (Xie et al., 2015) and employed the spacer with the lowest off-target efficiency for each promoter. The spacer sequences were added to the forward primer of each sgRNA using the p41sg (Zhao et al., 2008; Peng et al., 2018) plasmid as template; the linear plasmid containing different sgRNA fragments was amplified with primers sgRNA-P-F, sgRNA-B-F ∼ sgRNA-F-F, and sgRNA-R and then ligated with T4 DNA ligase with themselves, resulting pZJY41-sgRNA-P, pZJY41-sgRNA-B ∼ pZJY41-sgRNA-F plasmids. We obtained the sequence PpilA-spacerA-sgRNA by PCR from the plasmid pZJY41-sgRNA-A with the primer NdeI-F/R and then digested and connected it to the NdeI site of plasmid pZJY41-sgRNA-P to obtain two simultaneous expressions of sgRNA plasmid pZJY41-sgRNA-AP. Plasmids pZJY41-sgRNA-AB, pZJY41-sgRNA-APB, and pZJY41-sgRNA-DEF were constructed in the same way with different restriction sites.

### Construction of the CRISPRa-dCas9 System in *E. coli* and *M. xanthus*

We transformed plasmid pSWcuomxdCas9-ω into *E. coli* competent HB101 cells and obtained the pkk-CuOm strain. Then we transformed two plasmids into *E. coli* pkk-CuOm at the same time: pZJY41-sgRNA series plasmid carrying sgRNA with different spacer sequences and pKK232-Pepo series plasmid carrying an internal promoter and reporter gene *CAT*. These three plasmids all replicate autonomously in *E. coli*. Similarly, we constructed CRISPRa-dCas9 in *M. xanthus* by introducing plasmids pSWcuomxdCas9-ω and pZJY41-sgRNA. Notably, pSWcuomxdCas9-ω in *M. xanthus* was inserted at the *attB* site of the genome, while the plasmid pZJY41-sgRNA replicated autonomously.

### Extraction and Detection of Epothilones

ZE9 and mutants were grown overnight in 50 ml of CYE medium supplemented with Apra (25 μg/mL). The cultures were inoculated at a ratio of 2:100 into 50 mL of CMO medium containing 2% of XAD-16 resin. The resin was harvested with a strainer after 6 days and extracted with 3 mL of methanol by shaking at room temperature overnight. The supernatant was centrifuged for 10 min at 12,000 rpm and filtered with a 0.22-μm filter to remove the impurities. Twenty microliter of the sample was injected into a high-performance liquid chromatography (HPLC, SHIMADZU, Japan) system, analyzed on a Shim-pack MRC-ODS RP C18 column (4.6 mm × 250 mm, 4.60 μm; SHIMADZU, Japan), and monitored at 250 nm, with a mobile phase of 60% of methanol (HPLC grade) and 40% of H_2_O at a flow rate of 1.0 mL/min.

The major epothilone products are epothilones A and B. The structural difference between the two compounds is that there is an extra methyl on 12C in epothilone B (Supplementary Figure S4). Epothilones A and B are suggested to be produced by the nonspecific receiving of malonyl-CoA (producing epothilone A) or methyl-malonyl-CoA (producing epothilone B) by the second acyltransferase domain in EPOSC (Gerth et al., 2001). The yields of epothilones were quantified from the peak area in the UV chromatogram, by reference against a calibration standard. According to the elution time of standard samples, the peak of epothilone A appears at 12.5 min and that of epothilone B at 15 min.

### Transcriptional Analysis of Epothilone Genes With RT-qPCR

We collected samples continuously from the fermentation culture after 48 h of incubation. Then, total RNA of the samples was extracted using BIOZOL kits (Total RNA Extraction Reagent, BioFast, China) and then transcribed reversely into cDNA with the PrimeScript™ reagent kit with DNAase (Takara, Japan). The *gapA* gene (glyceraldehyde-3-phosphate dehydrogenase gene, MXAN_2815) was chosen as the reference gene for normalization. The transcriptional level of the epothilone gene cluster was analyzed by RT-qPCR on the LightCycler®480 system (Switzerland) with SYBR® Premix Ex Taq™ GC dye (Takara, Japan). All the primers used in RT-qPCR are listed in Supplementary Table S2.

## Results

### The Epothilone Gene Cluster Is a Big Operon Containing Multiple Internal Promoters

**FIGURE 1:**
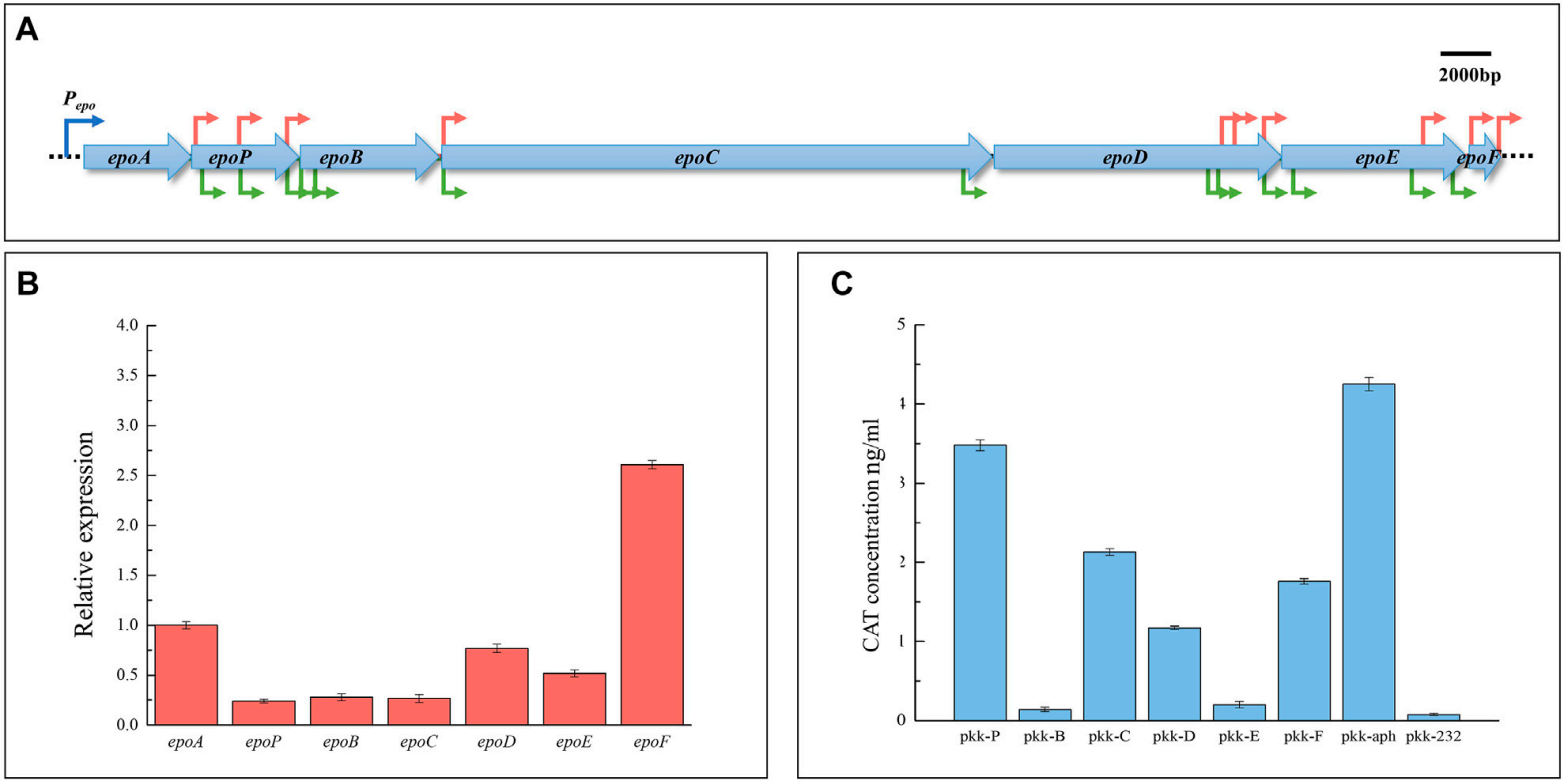
Internal promoters in the epothilone operon and their promotion activities. **(A)** Prediction of internal promoters in the epothilone operon from *S. cellulosum* So0157-2. The promoter prediction was performed using two online promoter prediction programs: “Neural Network Promoter Prediction” (red arrows) and BPROM (green arrows). **(B)** RT-qPCR analysis of expression levels of the seven operon genes in ZE9 after 48 h of incubation. The *epoA* expression was set as 1, and the expressions of the other six genes are shown as the relative expression of the *epoA* gene. **(C)** Activities of the separate internal promoters in *E. coli*. The activity of the reporter gene chloramphenicol acetyltransferase (CAT) was detected by using the CAT ELISA kit, with the *aphII* promoter as a positive control and the original plasmid pKK-232 as a negative control (no promoter upstream of the reporter gene *CAT*).

The epothilone biosynthetic gene cluster from *S. cellulosum* So0157-2 consists of seven ORFs transcribed in the same direction. These ORFs are shortly overlapped or separated with a short distance (15–147 bp), and no terminator structure was found in the intergenic regions (Figure 1A). The RT-PCR results showed that all the adjacent ORFs were co-transcribed (Supplementary Figure S5). Thus, the whole gene cluster is a huge operon. However, the transcriptional levels of the operon ORFs varied significantly. For example, *M. xanthus* ZE9 is an epothilone producer constructed with the whole epothilone gene cluster from *S. cellulosum* So0157-2 (Zhu et al., 2015). In ZE9, *epoP*, *epoB*, or *epoC* was transcribed about a quarter of the level of the first gene *epoA*, while the *epoD* transcription was 2-fold higher than that of the upstream *epoC*, and the last *epoF* had the highest expression level among all the operon genes, approximately 2.5 times that of *epoA* (Figure 1B). The differential expressions of operon genes suggested that additional promoters were probably present in the operon to coordinate the transcription of individual genes.

We searched for possible promoters in the epothilone operon using the “Neural Network Promoter Prediction” (https://www.fruitfly.org/seq_tools/promoter.html) and “BPROM” (http://www.softberry.com/berry.phtml) programs, both of which have been successfully employed for promoter prediction (Yang et al., 2013; Cebrián et al., 2014; Westholm et al., 2014; Umarov and Solovyev, 2017). The “Neural Network Promoter Prediction” program found that internal promoters occurred upstream of each of the operon genes, except for *epoD* (shown as red arrows in Figure 1A). Prediction with “BPROM” (http://www.softberry.com/berry.phtml) revealed similar results but excavated more potential promoters, including a promoter upstream of *epoD* (shown as green arrows in Figure 1A). These promoters were mostly located upstream of the initiation codons of downstream ORFs, and detailed information is provided in Supplementary Table S4.

To verify the transcriptional activities of internal promoters, we amplified 1000-bp junction regions between the adjacent operon ORFs as the promoter fragments, named P*epoP*, P*epoB* ~ P*epoF*. These regions included approximately 800 bp of the upstream gene and 200 bp of the downstream gene, which were amplified from *M. xanthus* ZE9 using the corresponding primer pairs (listed in Supplementary Table S2). These fragments, each containing at least one predicted internal promoter, were cloned into the pKK-232-8 plasmid, respectively, to control the expression of chloramphenicol acetyltransferase (CAT) (Supplementary Figure S1). The *aphII* promoter (Zhu et al., 2013) was constructed upstream of the *CAT* reporter gene as a positive control, and the pKK-232-8 plasmid containing the *CAT* gene with no promoter was used as a negative control. The activity of each internal promoter fragment was characterized by the CAT activity assay.

As shown in Figure 1C, the P*epoP* promoter had the highest activity, which was close to that of the *aphII* promoter, while the P*epoB* and P*epoE* promoters showed low activities, slightly higher than that of the negative control. The P*epoC*, P*epoD*, and P*epoF* promoters also exhibited remarkable transcriptional activities, which were lower than that of P*epoP*. Thus, multiple internal promoters are present in the epothilone operon. However, the detected promoter activities in *E. coli* were significantly inconsistent with the transcriptional levels of the genes in the epothilone operon. For instance, we did not find a strong promoter upstream of the *epoF*, which, however, displayed the highest transcriptional level among the ORFs in the epothilone operon. The results suggested that the functions of internal promoters might be complexly interfered in the operon.

### Operons and Separate Promoters Exhibit Different Transcriptional Activities

**FIGURE 2:**
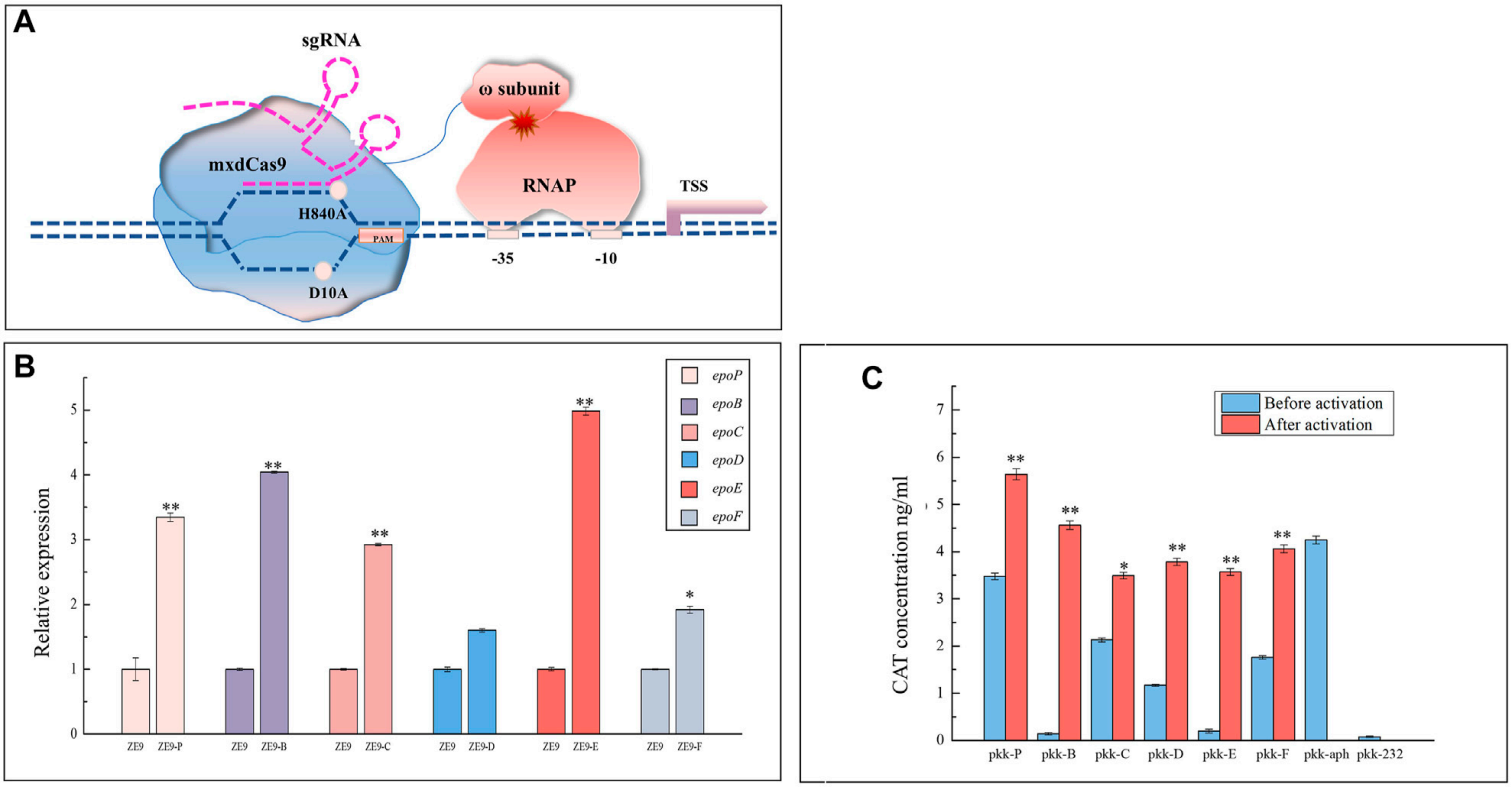
Activation of internal promoters. **(A)** Principle of CIRSPR-dCas9 activation. mxdCas9: codon-optimized inactivated dCas9 protein (H840A, D10A); ɷ subunit: the Omega subunit of RNA polymerase; RNAP: RNA polymerase; sgRNA: single guide RNA sequence, a combination of the CRISPR-associated RNA (crRNA) and the trans-activation crRNA (tracrRNA). **(B)** RT-qPCR analysis of expression levels of the six operon genes activated by internal promoters in different mutants of *M. xanthus* ZE9 after 48 h of incubation. The gene expressions in *M. xanthus* ZE9 were each set as 1, and the expressions of the operon genes in mutant strains are shown as the relative expression. **(C)** Activities of the separate internal promoters in *E. coli* before and after activation by CRISPRa. The CAT activities were detected with the *aphII* promoter as a positive control and the original plasmid pKK-232 (no promoter upstream of the reporter gene *CAT*). The *aphII* promoter was not activated. The error bars in **(B,C)** represent the standard deviation of three independent experiments. For statistical analysis between the ancestral strain and mutant strains, the signals of ** and * mean *p* < 0.01 and *p* < 0.05, respectively.

The CRISPR/dCas9 activation system (CRISPRa) is derived from the RNA-mediated CRISPR Cas system by fusing the nuclease-deficient Cas9 (dCas9) with a transcription activator, which combines with the sgRNA to guide the fused dCas9 protein to the target promoter to recruit more RNA polymerases and promote transcription (Bikard et al., 2013). CRISPRa has been used to activate gene expression in bacteria and fungi (Konermann et al., 2015; Peng et al., 2018; Cámara et al., 2020; Kiattisewee et al., 2021). To assay the effects of internal promoters on the transcription of operon genes, we performed in situ activation by introducing plasmids pSWcuomxdCas9-ω and pZJY41-sgRNA into the epothilone-producing *M. xanthus* ZE9. The pSWcuomxdCas9-ω plasmid carried the *dCas9-ω* gene encoding the dCas9 protein fused with the omega subunit of RNA polymerase (Peng et al., 2018) under the control of a cupric ion-induced promoter (Supplementary Figure S2), while the pZJY41-sgRNA plasmid carried an sgRNA targeting a specific promoter (Supplementary Figure S3). The principle of CIRSPR-dCas9 activation is shown in Figure 2A, and the sgRNA sequences targeting internal promoters are shown in Table 1. After incubation with cupric ions (200 μM), the transcriptional levels of the activated genes were significantly increased (*t*-test, *p* < 0.05), except for *epoD* (Figure 2B). According to the growth curves, the addition of cupric ions had no obvious influence on the growth of ZE9 or the activated mutants (Supplementary Figure S6).

**TABLE 1:** Information of the spacer sequences.

Name	Spacer	Coding strand	Off-target	Distance (bp)	Δg (kcals/mol)	Hairpin	GC%
**CuOm-P**	TCC​GGG​GGA​TGA​TGC​TCG​AG	+	8	−617	43.6	N	65.0
**CuOm-B**	TGA​GGA​GCC​TGT​TGC​AGA​AG	+	14	−376	38.2	N	55.0
**CuOm-C**	ACC​GTA​CCG​GCA​ACG​CTG​TTG	+	4	−165	45.3	N	61.9
**CuOm-D**	TGC​GGC​CGG​TAT​CCT​GGA​CGA	+	3	−117	47.8	N	66.7
**CuOm-E**	TGG​ATG​TAT​CCC​AAG​GTG​CT	+	2	−160	38.1	N	50.0
**CuOm-F**	AGC​TCT​TCT​TCC​GAA​ATG​CCG	+	2	−193	43.5	N	52.4

For comparison, we also constructed a CRISPRa system by transferring the pSWcuomxdCas9-ω and pZJY41-sgRNA plasmids into the above constructed *E. coli* strains to activate separate promoters of the epothilone operon. According to the CAT activity assay, the transcription activities of all these promoters were significantly improved (*t*-test, *p* < 0.05; Figure 2C). Consistent with the previous report (Bikard et al., 2013), weak promoters were activated more efficiently, and strong promoters were also activated but to low extents. For example, the transcription activity of the weakest promoter P*epoB* was increased by nearly 32-fold, while that of the strongest promoter P*epoP* was activated by approximately 60%. However, P*epoD* was activated by 260% in *E. coli*, but not significantly in situ activated in the operon in *M. xanthus*. The differences between the in situ activation in the operon and ectopic activation in a separate form suggested that the transcription activities of internal promoters were interfered in the operon.

### In Situ Activation of Internal Promoters Changes the Operon Transcriptional Profile

**FIGURE 3:**
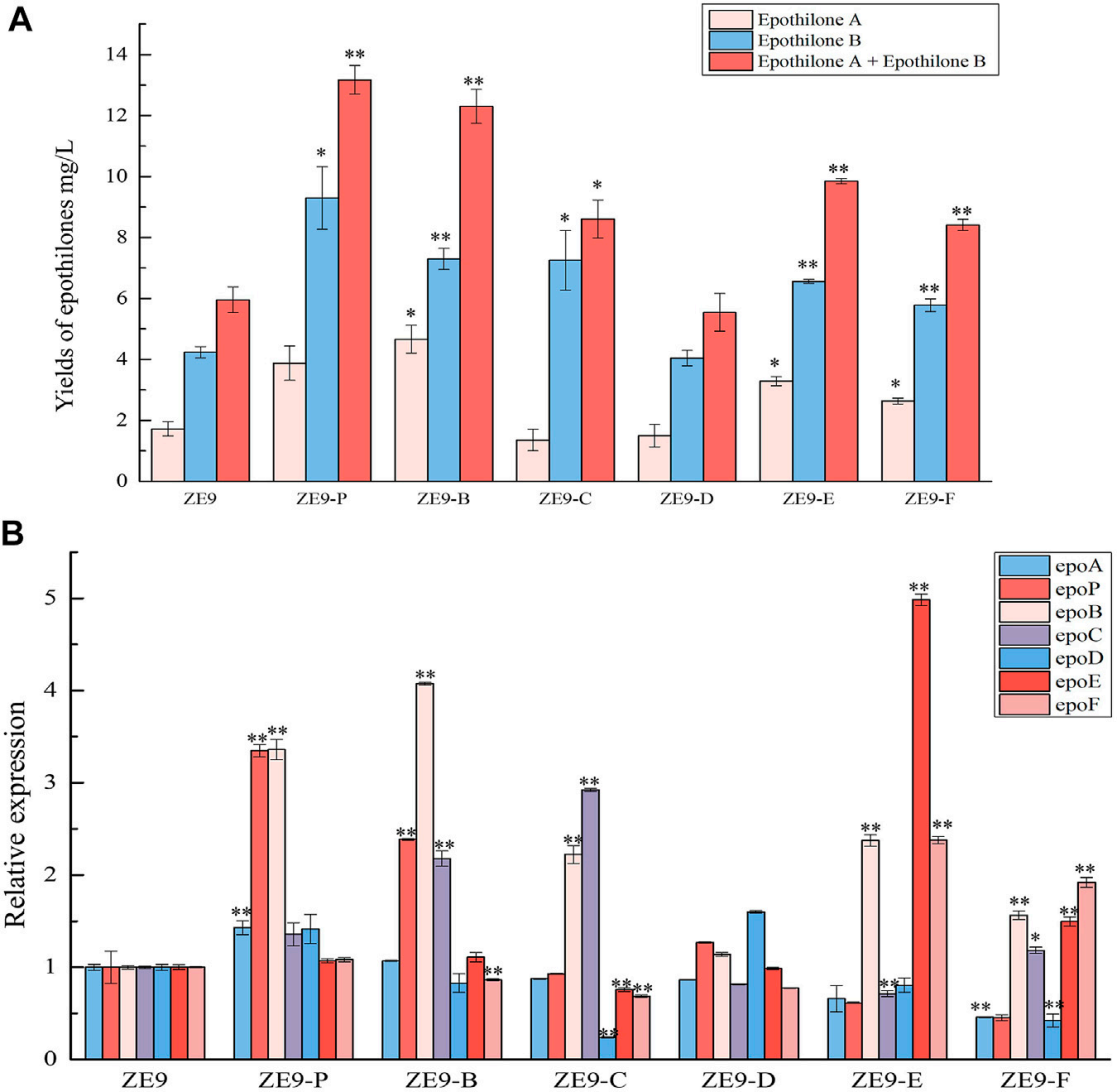
CRISPRa of internal promoters in the epothilone operon. **(A)** Yields of epothilones A and B and their summation in different activation mutants and *M. xanthus* ZE9. **(B)** RT-qPCR analysis of expression levels of the seven operon genes in different mutants and *M. xanthus* ZE9 after 48 h of incubation. The expressions of the operon genes in *M. xanthus* ZE9 were each set as 1, and the expressions of the genes in mutant strains are shown as the relative expression. The error bars represent the standard deviation of three independent experiments. For statistical analysis between the ancestral strain and mutant strains, the signals of ** and * mean *p* < 0.01 and *p* < 0.05, respectively.

We further assayed the yields of epothilones in these activation mutant strains. As shown in Figure 3A, activation of internal promoters also significantly increased the epothilone yields, except for that in ZE9-D, in which the expression of *epoD* failed to be activated. Comparably, the activation of P*epoC*, P*epoE,* and P*epoF* produced similar yields of epothilones, which, however, were lower than that from the activation of P*epoP* or P*epoB*. The result was inconsistent with the activation results of transcriptional levels, where the best activation was achieved on *epoE*, increasing by ∼ 4-fold compared to that in ZE9 (Figure 2B).

Furthermore, RT-qPCR analysis of all the operon genes in different activation mutants revealed that activating a specific internal promoter promoted the transcription of not only the target gene but also the nearby genes (Figure 3B). For example, when P*epoP* was activated, the transcriptional levels of *epoP*, the downstream *epoB*, and the upstream *epoA* were increased by 2.3-fold, 2.3-fold, and 43%, respectively. The activation of P*epoB* significantly promoted the transcription of *epoP*, *epoB*, and *epoC*. The downstream or upstream distant genes were also often affected by the activation of the promoters. For example, activation of P*epoC* resulted in a 1.9- and 1.2-fold transcriptional increase of *epoC* and *epoB* but decreased the transcription of *epoD*, *epoE*, and *epoF* by 76, 24, and 31%, respectively. Similarly, activating the posterior promoter (P*epoE* or P*epoF*) not only promoted the transcription of *epoE* or *epoF* genes but also affected the transcription of anterior genes of the operon, and even inhibited the first *epoA* gene. The above results suggested that in situ activation of internal promoters might change the operon transcriptional profile, thus leading to changes in epothilone yields.

### Combined Activation of Promoters for Further Increase of Transcription

**FIGURE 4:**
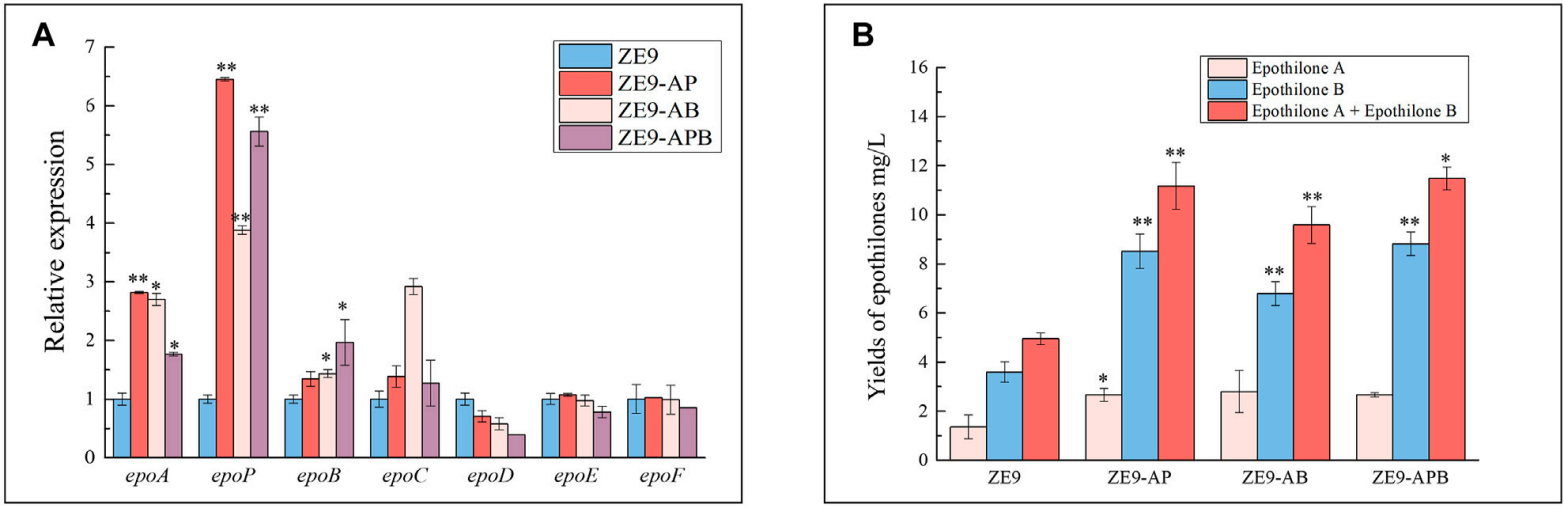
Combined activation of promoters in an operon. **(A)** RT-qPCR analysis of expression levels of the seven operon genes in the activation mutants after 48 h of incubation. The expressions of the seven operon genes in *M*. *xanthus* ZE9 were each set as 1, and the expressions of the genes in mutant strains are shown as the relative expression. **(B)** Yields of epothilone A and epothilone B in *M*. *xanthus* ZE9 and the activation mutants. The error bars represent the standard deviation of three independent experiments. For statistical analysis between the ancestral strain and mutant strains, the signals of ** and * mean *p* < 0.01 and *p* < 0.05, respectively.

Obviously, high yields of epothilones required an overall transcriptional increase of all the operon genes. In our previous work, activation of the starting promoter P*epoA* (the promoter of *epoA*) significantly increased the transcription of the operon genes and improved the yield of epothilones by 1.5-fold (Peng, et al., 2018). To further improve the transcriptional efficiency, we activated the P*epoA* and internal promoters together. Since independent activation of P*epoP* or P*epoB* resulted in relatively more epothilones (Figure 3A), we combined the activation of P*epoA* with P*epoP* or P*epoB* by introducing the plasmids pZJY41-sgRNA-AB and pZJY41-sgRNA-AP into ZE9-CuOm, a *M. xanthus* strain containing the plasmid pSWcuomxdCas9-ω. According to the RT-qPCR analysis, activating two promoters at the same time might lead to higher transcription levels of genes. For example, compared with that of ZE9, activation of P*epoP* alone increased the transcription levels of *epoA* and *epoP* by 1.4- and 2.3-fold, respectively, in ZE9-P (Figure 3B), but the combined activation of P*epoA* and P*epoP* improved the transcription levels of *epoA* and *epoP* by 1.8-fold and 6.5-fold, respectively, in ZE9-AP (Figure 4A). In ZE9-AB, compared with that of ZE9-B, combined activation of P*epoA* and P*epoB* produced higher transcription of *epoA*, *epoP*, and *epoC* but lower transcription of *epoB*. Similar to that in ZE9-P or ZE9-B, combined activation of P*epoA* with P*epoP* or P*epoB* did not increase the transcription of the posterior genes in the operon.

Corresponding to the transcription of the epothilone operon, the production abilities of epothilones were improved similarly in ZE9-AP and ZE9-AB: The epothilone yields were increased from 4.95 mg/L in ZE9 to 11.17 mg/L in ZE9-AP and 9.59 mg/L in ZE9-AB (Figure 4B). No significant difference was found between ZE9-AP and ZE9-AB. However, compared with ZE9-P, in which the P*epoP* was activated alone resulting in a 1.17-fold improvement of epothilone yield, further increase in transcription of the epothilone operon did not lead to a higher yield in ZE9-AP. The potential reason is that transcription of the posterior genes was not efficiently activated by the activation combination. Notably, further combined activation of P*epoA*, P*epoP*, and P*epoB* did not lead to higher transcription of the anterior genes in the operon and neither increased the transcription of the posterior genes, thus leading to similar epothilone production in the ZE9-AP, ZE9-AB, and ZE9-APB strains (Figure 4).

### Better Effects of Promoter Activation in Low Epothilone-Producing Strains

**FIGURE 5:**
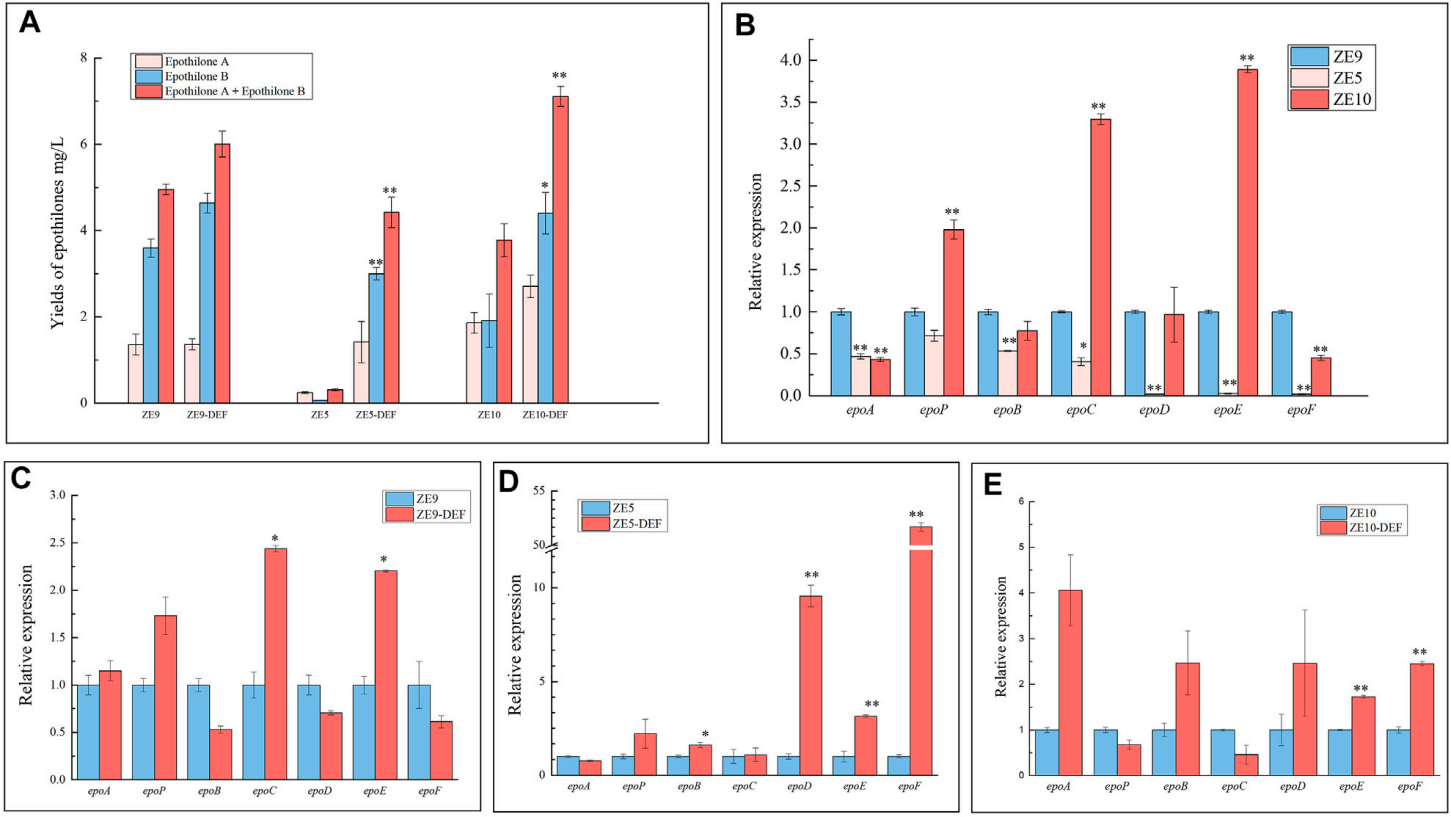
CRISPRa of internal promoters in different epothilone-producing *M*. *xanthus* strains ZE9, ZE5, and ZE10. **(A)** Yield of epothilones in wild strains and the activation mutants. **(B)** RT-qPCR analysis of expression levels of the seven operon genes in ZE9, ZE5, and ZE10 after 48 h of incubation. The expressions of the seven operon genes in *M*. *xanthus* ZE9 were each set as 1, and the expressions of the genes in other strains are shown as the relative expression. **(C,D)** RT-qPCR analysis of expression levels of the seven operon genes in the activation mutants after 48 h of incubation. The expressions of the seven operon genes in wild strains were each set as 1, and the expressions of the genes in mutant strains are shown as the relative expression. The error bars represent the standard deviation of three independent experiments. For statistical analysis between the ancestral strain and mutant strains, the signals of ** and * mean *p* < 0.01 and *p* < 0.05, respectively.

We previously constructed dozens of epothilone-producing *M. xanthus* strains, in which the epothilone biosynthetic gene cluster was inserted into different sites of the DZ2 genome, producing mutants with varied epothilone production abilities (Zhu et al., 2015). We also combined promoter activation in three *M. xanthus* strains with different transcription levels of the epothilone genes, that is, ZE9, ZE5, and ZE10. Among these three strains, ZE9 had the highest epothilone production ability, followed by ZE10 and ZE5 exhibiting the lowest epothilone yields (Figure 5A). Consistently, the transcriptional levels of the seven ORFs in ZE5 were mostly lower than those of the other two strains (Figure 5B). Specifically in ZE10, *epoP*, *epoC*, and *epoE* were transcribed at higher levels, but *epoA* and *epoF* were significantly lower than that in ZE9. In ZE5, the operon genes were transcribed at much lower levels than that in ZE9, especially the posterior genes in the operon. The transcription levels of *epoD*, *epoE*, and *epoF* in ZE5 were only 1–2% of those in ZE9, which were evidently the limitation for the expression of the whole gene cluster.

We combined the activation on the posterior promoters (P*epoD*, P*epoE*, and P*epoF*) in these epothilone-producing strains. As expected, the epothilone yields were all increased in these strains, and the highest 15-fold increase of epothilone yield was obtained in ZE5 with the DEF promoter activation (Figure 5A). Consistent with the yields of epothilones, the highest activation efficiency also occurred in the ZE5-DEF strain (the relative transcriptional increases in the three strains are shown in Figures 5C–E). In ZE5-DEF, the transcriptional levels of the three activated genes *epoD*, *epoE*, and *epoF* were increased by 9.6, 3.1, and 51.7 times, respectively, and *epoP* and *epoB* were also increased slightly. However, the transcriptional changes in operon genes suggested that the interferences between operon promoters were complex. For example, significantly increased transcription occurred with *epoC* and *epoE* in the ZE9-DEF strain, *epoE* and *epoF* in ZE10-DEF, and *epoD*, *epoE*, and *epoF* in ZE5-DEF. The transcription of the anterior genes was mostly unchanged significantly. Comparably, while the transcription of operon genes and yield of epothilones in ZE9 were both higher than those of ZE5 and ZE10, the activation efficiency in ZE9-DEF was much lower. Seemingly consistent with the above activation results of separate promoters, activation of weak promoters in lower epothilone-producing strains produces better activation effects.

## Discussion

Transcription regulation is always a topic of biological concern. Operons, which are only present in prokaryotes, are clusters of genes that share the same promoter and are transcribed as a single large mRNA that contains multiple structural genes or cistrons. Bacteria have established multiple mechanisms to ensure relative expressional levels of individual genes in operons to meet the requirements of cells and environments, including internal promoter regulation (Reznikoff, 1992; Huang et al., 2007; Shin and Price, 2007). The transcriptional interference between tandem promoters is recognized as a potentially widespread mechanism to regulate gene expression (Shearwin et al., 2005; Palmer et al., 2011). There are many studies on the regulation of single internal promoters in the expression of operon genes, but a few studies have been performed on transcriptional interferences between multiple operon promoters. For example, the 14-kb *CAP1* gene cluster in *Staphylococcus aureus* is transcriptionally controlled by a strong upstream promoter and five weak internal promoters, and the internal promoters showed significant activities only after removing the primary promoter (Ouyang and Lee, 1997). In the cyanobacterium *Anabaena* sp. strain PCC 7120, a zinc-responsive operon contains four distinct promoters induced by metal depletion; the upstream two were directly targeted by a Zur regulator, but the four internal promoters were constitutively derepressed in a *zur* mutant, indicating that all the internal promoters interfere with each other (Mauro et al., 2013).

The internal promoters are widely present in prokaryotic and eukaryotic cells and may have indispensable functions. In human spuma retrovirus (HSRV), an internal promoter was found in the HSRV operon, which was constitutively activated by the internal promoter and thus resulted in the accumulation of nonstructural proteins inside the host cell at the early stage of HSRV replication (Löchelt et al., 1993; Xu et al., 2019). This kind of internal promoters has been reported for wide use in the construction of retroviral vectors, in which the internal promoter avoids the problems associated with the sequences in the intron that can interfere with splicing the message for the second gene (Albagli et al., 2019). In the halophilic archaeon *Haloferax volcanii*, the *rpl37R* gene coding ribosomal L37. eR protein is overlapped and co-transcribed with an upstream gene, and the two genes are regulated independently by an internal promoter located in the upstream ORF, ensuring their strong expression in the exponential growth phase (Maier et al., 2015). An internal promoter P_4532_ was found in the type VI secretion system (T6SS) gene cluster in enteroaggregative *E. coli* cells; the transcription of T6SS was regulated by the main promoter, and the internal promoter allowed the optimum production of T6SS under the condition where enteroaggregative *E. coli* encounters competing species (Brunet et al., 2020). In homo genome, the aberrant expression of PRDM8 (PR domain containing 8) is closely related to down syndrome (DS); the PRDM8 is transcribed as two different transcripts, which are regulated by an internal promoter in PRDM8, leading to a significantly improved expression of transcript variant two in DS patients (Lu et al., 2016). Taken together, internal promoters normally play a complementary role for gene expression, thus regulating viral infection, bacterial growth and metabolisms, and human diseases.

In this study, we demonstrated that the big epothilone gene operon contained multiple internal promoters. These internal promoters exhibited different transcriptional activities in the operon and separate forms. In *E. coli*, these internal promoters were constructed in different mutants and thus independently regulate the expression of the reporter gene *CAT* in a separated form. In *M. xanthus*, these internal promoters exist in the operon and may be subject to more complex regulation. The potential mechanisms for the different transcriptional activities of promoters are complex. It has been reported that upstream promoters can block the activities of downstream promoters (Namprachan-Frantz et al., 2014), and some internal promoters are active under certain conditions (Seidl et al., 2014). However, in addition to the interference between internal promoters in the operon, some unknown regulators might also influence the gene transcription. Obviously, internal promoters in the epothilone gene cluster were recognized and regulated in heterologous hosts, and there should be some regulatory proteins involved, such as a sigma factor or transcriptional regulator. Thus, although the same CRISPRa system was used for each promoter, the regulatory networks in *E. coli* and *M. xanthus* may have different effects on the activation efficiency.

In previous studies, researchers found that promoters with different transcriptional activities were activated at different degrees with the CRISPR activation: The best activation was obtained with weak promoters, and the relative activation effects would diminish as the promoter becomes stronger (Bikard et al., 2013). Similarly, in our study, weak separate promoters were easily activated, while stronger promoters were less activated. Furthermore, if the promoters were in operons, the CRISPR activation was at a higher efficiency in the low-transcription operons than that in the high-expression operons. For example, the *epoD*, *epoE*, and *epoF* in ZE5 were transcribed at much lower levels than those in ZE9 or ZE10 and evidently became the short board for operon transcription. The CRISPR activation in ZE5 led to 3 ∼ 50-fold transcriptional improvement of these genes and about 14.7-fold increase of the epothilone yield; the effect is more significant than those on ZE9 and ZE10. Besides, the transcription by internal promoters seems to interfere with each other. For example, when the P*epoP* promoter was activated, the transcription levels of *epoP* and downstream *epoB* were increased by 2.5-fold and 1.5-fold, respectively. In ZE9-B, after activation of P*epoB*, the expression of not only *epoB* but also the downstream *epoC* and the upstream *epoP* was markedly activated. Thus, we suggested that internal promoters, together with the starting promoter, complexly coordinate the transcriptional processes of genes in big operons for the product yield to meet the environmental requirement.

Simultaneous transcription of tandem or convergent (face-to-face) arrangements of promoters might lead to interactions between RNA polymerases (RNAPs), which causes transcriptional interferences and is often with important consequences for gene expression (Callen et al., 2004). In such cases, transcription from one promoter can have a significant inhibitory effect on the transcription from other promoters, often with important regulatory consequences (Prescott and Proudfoot, 2002; Martens et al., 2004). In bacteria, the transcriptional interferences may result from three mechanisms: occlusion (in which passing RNAPs block the access to the following promoters), collisions between elongating RNAPs, and sitting duck interference (in which RNAP complexes waiting to fire at the promoter are removed by passing RNAPs) (Sneppen et al., 2005). In addition to the aforementioned mechanisms, Leng et al. reported that the transcription by RNAPs can also stimulate localized DNA supercoiling, which blocks the transcription initiation of the downstream promoter in *E. coli* cells (Leng and McMacken, 2002). Moreover, internal promoters in operons may respond to multiple stimuli, thus being different from the starting promoter (Mauro et al., 2013). Therefore, the transcriptional interference between tandem promoters is not totally satisfactorily explained by RNAP interactions—there should be other unknown regulatory patterns.

The inconsistency in the transcriptional levels of operon genes often limits the yield of secondary metabolites. The inconsistent expression levels of genes in operons observed in different bacterial species are challenging to the concept of operons (Kaebernick et al., 2000; Yoon and Golden, 2001) and also impede our engineering work to control the transcription of operon genes. Our results presented in this study indicated that multiple internal promoters are present in the epothilone gene cluster, and the transcriptional processes of these internal promoters may intricately interfere with each other. Although little is known of the mechanism involved, regulation of operon internal promoters should be crucial for the biosynthetic pathways of secondary metabolites encoded by a big operon. Tuning the transcriptional activities of operon promoters, such as using the CRISPRa technique, can efficiently improve the metabolite yields.

## Data Availability

The original contributions presented in the study are included in the article/Supplementary Material. Further inquiries can be directed to the corresponding authors.

## References

[B1] Albagli O. Maugein A. Huijbregts L. Bredel D. Carlier G. Martin P. (2019). New α- and SIN γ-Retrovectors for Safe Transduction and Specific Transgene Expression in Pancreatic β Cell Lines. BMC Biotechnol. 19 (1), 35. 10.1186/s12896-019-0531-9 31208395 PMC6580483

[B2] Bikard D. Jiang W. Samai P. Hochschild A. Zhang F. Marraffini L. A. (2013). Programmable Repression and Activation of Bacterial Gene Expression Using an Engineered CRISPR-Cas System. Nucleic Acids Res. 41 (15), 7429–7437. 10.1093/nar/gkt520 23761437 PMC3753641

[B3] Bollag D. M. McQueney P. A. Zhu J. Hensens O. Koupal L. Liesch J. (1995). Epothilones, a New Class of Microtubule-Stabilizing Agents With a Taxol-Like Mechanism of Action. Cancer Res. 55 (11), 2325–2333. 10.1007/s002620050191 7757983

[B4] Brunet Y. R. Bernard C. S. Cascales E. (2020). Fur-dam Regulatory Interplay at an Internal Promoter of the Enteroaggregative *Escherichia coli* Type VI Secretion *Sci1* Gene Cluster. J. Bacteriol. 202 (10). 10.1128/JB.00075-20 PMC7186456 32152218

[B5] Callen B. P. Shearwin K. E. Egan J. B. (2004). Transcriptional Interference Between Convergent Promoters Caused by Elongation Over the Promoter. Mol. Cel. 14 (5), 647–656. 10.1016/j.molcel.2004.05.010 15175159

[B6] Cámara E. Lenitz I. Nygård Y. (2020). A CRISPR Activation and Interference Toolkit for Industrial *Saccharomyces Cerevisiae* Strain KE6-12. Sci. Rep. 10 (1), 14605. 10.1038/s41598-020-71648-w 32884066 PMC7471924

[B7] Cebrián R. Rodríguez-Ruano S. Martínez-Bueno M. Valdivia E. Maqueda M. Montalbán-López M. (2014). Analysis of the Promoters Involved in Enterocin AS-48 Expression. Plos One. 9 (3), e90603. 10.1371/journal.pone.0090603 24594763 PMC3942455

[B8] de Hoon M. Makita Y. Nakai K. Miyano S. (2005). Prediction of Transcriptional Terminators in *Bacillus Subtilis* and Related Species. Plos Comp. Biol. preprint (3), e25. 10.1371/journal.pcbi.0010025.eor PMC1187862 16110342

[B9] Gerth K. Bedorf N. Höfle G. Irschik H. Reichenbach H. (1996). Epothilons A and B: Antifungal and Cytotoxic Compounds From Sorangium Cellulosum (Myxobacteria). Production, Physico-Chemical and Biological Properties. J. Antibiot. 49 (6), 560–563. 10.7164/antibiotics.49.560 8698639

[B10] Gerth K. Steinmetz H. Höfle G. Reichenbach H. (2001). Studies on the Biosynthesis of Epothilones. The PKS and Epothilone C/D Monooxygenase. J. Antibiot. 54 (2), 144–148. 10.7164/antibiotics.54.144 11302486

[B11] Güell M. van Noort V. Yus E. Chen W.-H. Leigh-Bell J. Michalodimitrakis K. (2009). Transcriptome Complexity in a Genome-Reduced Bacterium. Science. 326 (5957), 1268–1271. 10.1126/science.1176951 19965477

[B12] Han K. Li Z.-f. Peng R. Zhu L.-p. Zhou T. Wang L.-g. (2013). Extraordinary Expansion of a *Sorangium Cellulosum* Genome From an Alkaline Milieu. Sci. Rep. 3 (1), e1000565–1289. 10.1038/srep02101 PMC3696898 23812535

[B13] Huang P. Pleasance E. D. Maydan J. S. Hunt-Newbury R. O'Neil N. J. Mah A. (2007). Identification and Analysis of Internal Promoters in *Caenorhabditis Elegans* Operons. Genome Res. 17 (10), 1478–1485. 10.1101/gr.6824707 17712020 PMC1987351

[B14] Jones A. C. Gerwick L. Gonzalez D. Dorrestein P. C. Gerwick W. H. (2009). Transcriptional Analysis of the Jamaicamide Gene Cluster From the Marine Cyanobacterium *Lyngbya Majuscula* and Identification of Possible Regulatory Proteins. BMC Microbiol. 9 (1), 247. 10.1186/1471-2180-9-247 19951434 PMC2799420

[B15] Julien B. Shah S. Ziermann R. Goldman R. Katz L. Khosla C. (2000). Isolation and Characterization of the Epothilone Biosynthetic Gene Cluster from *Sorangium Cellulosum* . Gene. 249 (1), 153–160. 10.1016/S0378-1119(00)00149-9 10831849

[B16] Kaebernick M. Dittmann E. Börner T. Neilan B. A. (2002). Multiple Alternate Transcripts Direct the Biosynthesis of Microcystin, a Cyanobacterial. Appl. Environ. Microbiol. 68 (2), 449–455. 10.1128/AEM.68.2.449-455.2002 11823177 PMC126702

[B17] Kaebernick M. Neilan B. A. Börner T. Dittmann E. (2000). Light and the Transcriptional Response of the Microcystin Biosynthesis Gene Cluster. Appl. Environ. Microbiol. 66 (8), 3387–3392. 10.1128/AEM.66.8.3387-3392.2000 10919796 PMC92160

[B18] Kiattisewee C. Dong C. Fontana J. Sugianto W. Peralta-Yahya P. Carothers J. M. (2021). Portable Bacterial CRISPR Transcriptional Activation Enables Metabolic Engineering in *Pseudomonas Putida* . Metab. Eng. 66, 283–295. 10.1016/j.ymben.2021.04.002 33930546

[B19] Konermann S. Brigham M. D. Trevino A. E. Joung J. Abudayyeh O. O. Barcena C. (2015). Genome-Scale Transcriptional Activation by an Engineered CRISPR-Cas9 Complex. Nature. 517 (7536), 583–588. 10.1038/nature14136 25494202 PMC4420636

[B20] Leng F. McMacken R. (2002). Potent Stimulation of Transcription-Coupled DNA Supercoiling by Sequence-Specific DNA-Binding Proteins. Proc. Natl. Acad. Sci. 99 (14), 9139–9144. 10.1073/pnas.142002099 12093906 PMC123107

[B21] Löchelt M. Muranyi W. Flügel R. M. (1993). Human Foamy Virus Genome Possesses an Internal, Bel-1-Dependent and Functional Promoter. Proc. Natl. Acad. Sci. 90 (15), 7317–7321. 10.1073/pnas.90.15.7317 8394017 PMC47128

[B22] Lu Z. Liu Y. Ren Z. Yan J. (2016). PRDM8 Internal Promoter Hyperhydroxymethylation Correlates With Increased Expression of the Corresponding Transcript in Down Syndrome. Mol. Med. Rep. 14 (2), 1227–1234. 10.3892/mmr.2016.5375 27278638

[B23] Ma J.-C. Newman A. J. Hayward R. S. (1981). Internal Promoters of the *rpoBC* Operon of *Escherichia coli* . Mol. Gen. Genet. 184 (3), 548–550. 10.1007/BF00352538 6278264

[B24] Maier L.-K. Benz J. Fischer S. Alstetter M. Jaschinski K. Hilker R. (2015). Deletion of the Sm1 Encoding Motif in the *Lsm* Gene Results in Distinct Changes in the Transcriptome and Enhanced Swarming Activity of *Haloferax* Cells. Biochimie. 117, 129–137. 10.1016/j.biochi.2015.02.023 25754521

[B25] Makita Y. Nakao M. Ogasawara N. Nakai K. (2004). DBTBS: Database of Transcriptional Regulation in *Bacillus Subtilis* and its Contribution to Comparative Genomics. Nucleic Acids Res. 32 (90001), 75D–77D. 10.1093/nar/gkh074 PMC308808 14681362

[B26] Martens J. A. Laprade L. Winston F. (2004). Intergenic Transcription Is Required to Repress the *Saccharomyces Cerevisiae SER3* Gene. Nature. 429 (6991), 571–574. 10.1038/nature02538 15175754

[B27] Molnár I. Schupp T. Ono M. Zirkle R. Milnamow M. Nowak-Thompson B. (2000). The Biosynthetic Gene Cluster for the Microtubule-Stabilizing Agents Epothilones A and B from *Sorangium Cellulosum* So Ce90. Chem. Biol. 7 (2), 97–109. 10.1016/S1074-5521(00)00075-2 10662695

[B28] Murakawa G. J. Kwan C. Yamashita J. Nierlich D. P. (1991). Transcription and Decay of the Lac Messenger: Role of an Intergenic Terminator. J. Bacteriol. 173 (1), 28–36. 10.1128/jb.173.1.28-36.1991 1702782 PMC207152

[B29] Namprachan-Frantz P. Rowe H. M. Runft D. L. Neely M. N. (2014). Transcriptional Analysis of the *Streptococcus Pyogenes* Salivaricin Locus. J. Bacteriol. 196 (3), 604–613. 10.1128/JB.01009-13 24244008 PMC3911159

[B30] Napolitano M. Rubio M. A. Camargo S. Luque I. (2013). Regulation of Internal Promoters in a Zinc-Responsive Operon Is Influenced by Transcription from Upstream Promoters. J. Bacteriol. 195 (6), 1285–1293. 10.1128/JB.01488-12 23316045 PMC3591996

[B31] Osbourn A. E. Field B. (2009). Operons. Cell. Mol. Life Sci. 66, 3755–3775. 10.1007/s00018-009-0114-3 19662496 PMC2776167

[B32] Ouyang S. Lee C. Y. (1997). Transcriptional Analysis of Type 1 Capsule Genes in *Staphylococcus Aureus* . Mol. Microbiol. 23 (3), 473–482. 10.1046/j.1365-2958.1997.d01-1865.x 9044281

[B33] Palmer A. C. Egan J. B. Shearwin K. E. (2011). Transcriptional Interference by RNA Polymerase Pausing and Dislodgement of Transcription Factors. Transcription. 2 (1), 9–14. 10.4161/trns.2.1.13511 21326903 PMC3023640

[B34] Peng R. Wang Y. Feng W.-w. Yue X.-j. Chen J.-h. Hu X.-z. (2018). CRISPR/dCas9-Mediated Transcriptional Improvement of the Biosynthetic Gene Cluster for the Epothilone Production in *Myxococcus Xanthus* . Microb. Cel Fact. 17 (1), 15. 10.1186/s12934-018-0867-1 PMC5787926 29378572

[B35] Prescott E. M. Proudfoot N. J. (2002). Transcriptional Collision between Convergent Genes in Budding Yeast. Proc. Natl. Acad. Sci. 99 (13), 8796–8801. 10.1073/pnas.132270899 12077310 PMC124378

[B36] Reznikoff W. S. (1992). The Lactose Operon-Controlling Elements: a Complex Paradigm. Mol. Microbiol. 6 (17), 2419–2422. 10.1111/j.1365-2958.1992.tb01416.x 1328815

[B37] Seidl M. D. Nunes F. Fels B. Hildebrandt I. Schmitz W. Schulze‐Osthoff K. (2014). A Novel Intronic Promoter of the *Crem* Gene Induces Small ICER (smICER) Isoforms. FASEB j. 28 (1), 143–152. 10.1096/fj.13-231977 24022402

[B38] Sharma C. M. Hoffmann S. Darfeuille F. Reignier J. Findeiß S. Sittka A. (2010). The Primary Transcriptome of the Major Human Pathogen *Helicobacter Pylori* . Nature. 464 (7286), 250–255. 10.1038/nature08756 20164839

[B39] Shearwin K. Callen B. Egan J. (2005). Transcriptional Interference - a Crash Course. Trends Genet. 21, 339–345. 10.1016/j.tig.2005.04.009 15922833 PMC2941638

[B40] Shin J.-H. Price C. W. (2007). The SsrA-SmpB Ribosome rescue System Is Important for Growth of *Bacillus Subtilis* at Low and High Temperatures. J. Bacteriol. 189 (10), 3729–3737. 10.1128/JB.00062-07 17369301 PMC1913333

[B41] Sneppen K. Dodd I. B. Shearwin K. E. Palmer A. C. Schubert R. A. Callen B. P. (2005). A Mathematical Model for Transcriptional Interference by RNA Polymerase Traffic in *Escherichia coli* . J. Mol. Biol. 346, 399–409. 10.1016/j.jmb.2004.11.075 15670592

[B42] Umarov R. K. Solovyev V. V. (2017). Recognition of Prokaryotic and Eukaryotic Promoters Using Convolutional Deep Learning Neural Networks. Plos One. 12 (2): e0171410. 10.1371/journal.pone.0171410 28158264 PMC5291440

[B43] Westholm J. Miura O. P. Miura P. Olson S. Shenker S. Joseph B. (2014). Genome-Wide Analysis of Drosophila Circular RNAs Reveals Their Structural and Sequence Properties and Age-Dependent Neural Accumulation. Cel Rep. 9 (5), 1966–1980. 10.1016/j.celrep.2014.10.062 PMC4279448 25544350

[B44] Xie S. S. Zhang Y. Zhang L. S. Li G. L. Zhao C. Z. Ni P. (2015). sgRNA Design for the CRISPR/Cas9 System and Evaluation of its Off-Target Effects. Yi Chuan. 37 (11), 1125–1136. 10.16288/j.yczz.15-093 26582526

[B45] Xu S. Yang W. Yuan P. Yan J. Tang Y. Zheng Y. (2019). The Long-Noncoding RNA Lnc-NONH Enhances the Early Transcription of Prototype Foamy Virus via Upregulating Expression of miR-34c-5p and Tas Protein. Intervirology. 62 (3-4), 156–163. 10.1159/000502038 31430761

[B46] Yang M. Zhang W. Ji S. Cao P. Chen Y. Zhao X. (2013). Generation of an Artificial Double Promoter for Protein Expression in *Bacillus Subtilis* Through a Promoter Trap System. Plos One. 8 (2), e56321. 10.1371/journal.pone.0056321 23409173 PMC3568030

[B47] Yoon H.-S. Golden J. W. (2001). PatS and Products of Nitrogen Fixation Control Heterocyst Pattern. J. Bacteriol. 183 (8), 2605–2613. 10.1128/JB.183.8.2605-2613.2001 11274121 PMC95178

[B48] Yue X.-j. Cui X.-w. Zhang Z. Hu W.-f. Li Z.-f. Zhang Y.-m. (2018). Effects of Transcriptional Mode on Promoter Substitution and Tandem Engineering for the Production of Epothilones in *Myxococcus Xanthus* . Appl. Microbiol. Biotechnol. 102 (13), 5599–5610. 10.1007/s00253-018-9023-4 29705958 PMC5999154

[B49] Yue X.-j. Cui X.-w. Zhang Z. Peng R. Zhang P. Li Z.-f. (2017). A Bacterial Negative Transcription Regulator Binding on an Inverted Repeat in the Promoter for Epothilone Biosynthesis. Microb. Cel Fact. 16 (1), 92. 10.1186/s12934-017-0706-9 PMC5442856 28535774

[B50] Zhao J.-y. Zhong L. Shen M.-j. Xia Z.-j. Cheng Q.-x. Sun X. (2008). Discovery of the Autonomously Replicating Plasmid pMF1 From *Myxococcus Fulvus* and Development of a Gene Cloning System in *Myxococcus Xanthus* . Appl. Environ. Microbiol. 74 (7), 1980–1987. 10.1128/aem.02143-07 18245244 PMC2292591

[B51] Zhu L.-P. Li Z.-F. Sun X. Li S.-G. Li Y.-Z. (2013). Characteristics and Activity Analysis of Epothilone Operon Promoters From *Sorangium Cellulosum* Strains in *Escherichia coli* . Appl. Microbiol. Biotechnol. 97 (15), 6857–6866. 10.1007/s00253-013-4830-0 23549746

[B52] Zhu L.-P. Yue X.-J. Han K. Li Z.-F. Zheng L.-S. Yi X.-N. (2015). Allopatric Integrations Selectively Change Host Transcriptomes, Leading to Varied Expression Efficiencies of Exotic Genes in *Myxococcus Xanthus* . Microb. Cel Fact. 14 (1), 105. 10.1186/s12934-015-0294-5 PMC4509775 26194479

