# Supplementary figures and images for: Internal Promoters and Their Effects on the Transcription of Operon Genes for Epothilone Production in Myxococcus xanthus
Source: Front Bioeng Biotechnol. 2021 Oct 27;9:758561. doi: 10.3389/fbioe.2021.758561 (PMC8579030; doi:10.3389/fbioe.2021.758561)

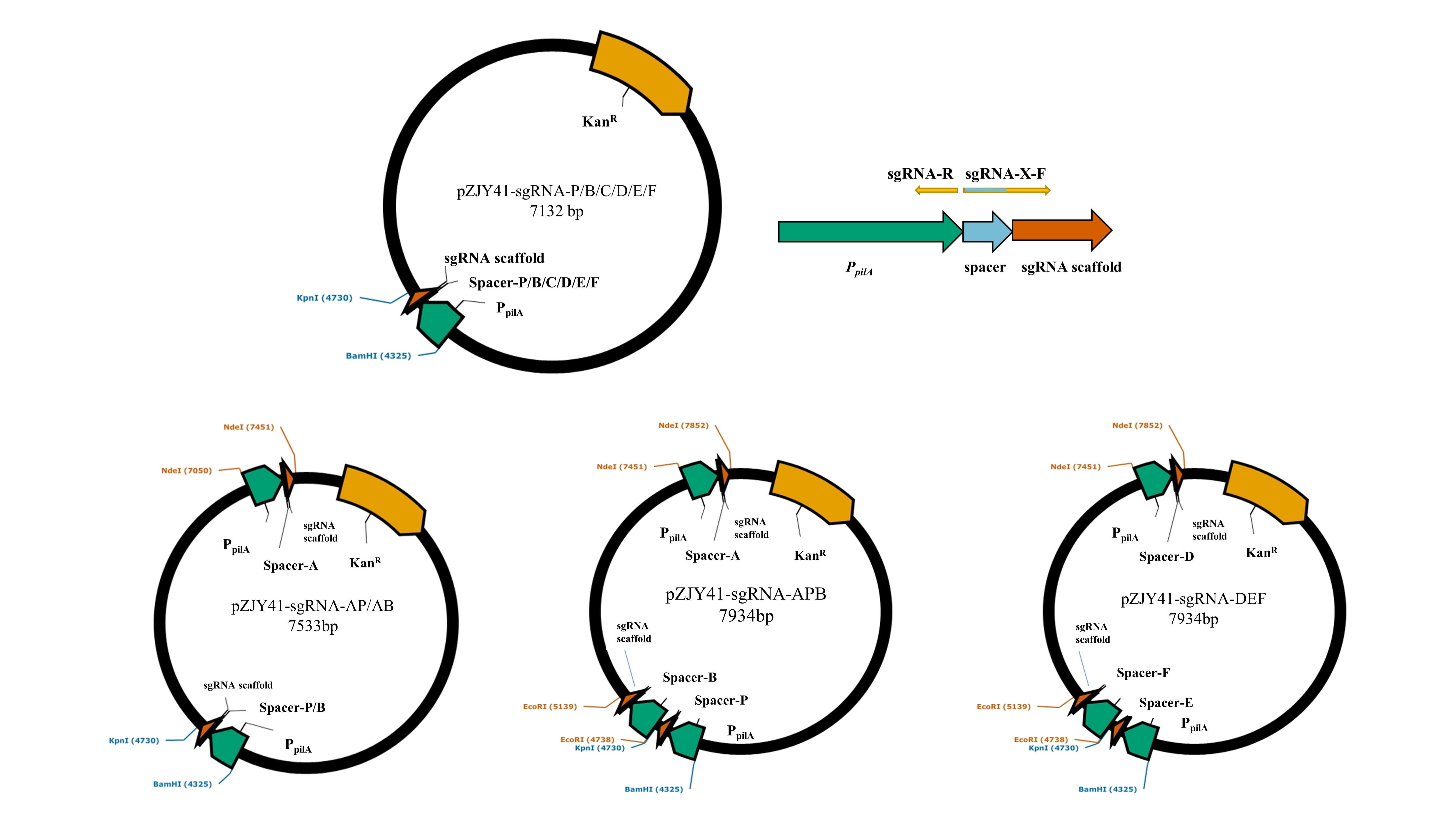

Supplement: Supplementary file 1 [file Image3.JPEG]

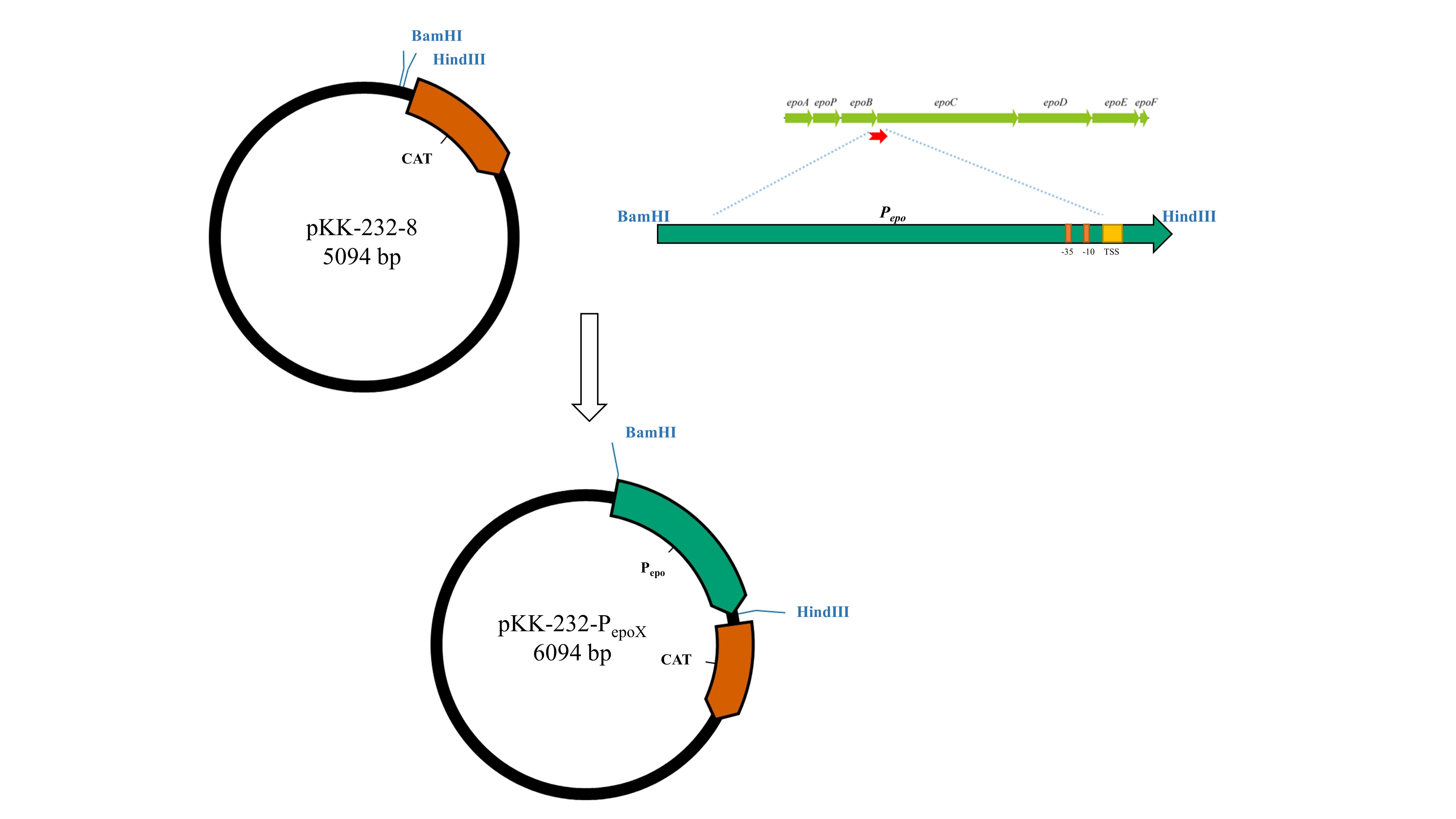

Supplement: Supplementary file 3 [file Image1.JPEG]

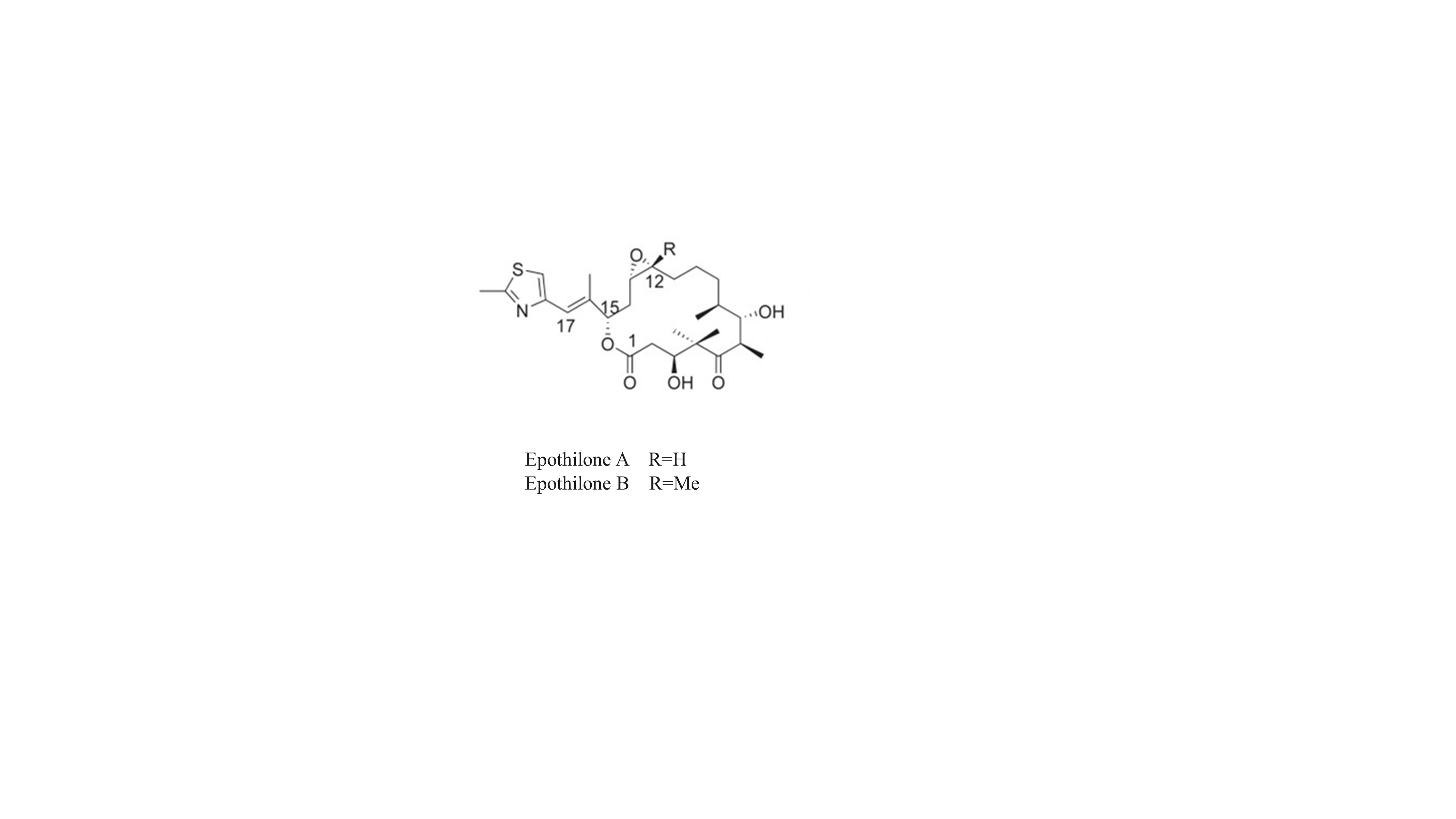

Supplement: Supplementary file 4 [file Image4.JPEG]

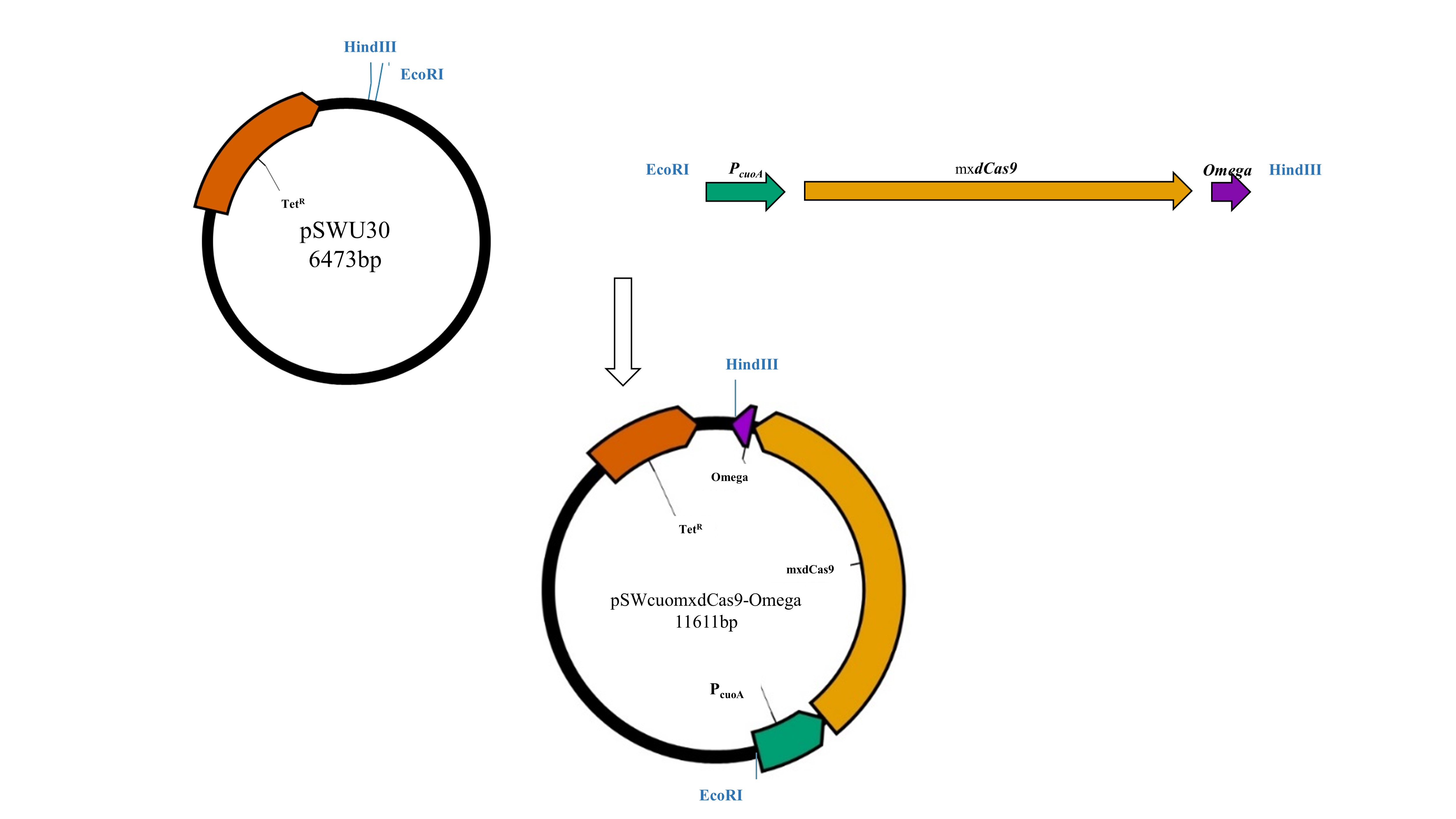

Supplement: Supplementary file 5 [file Image2.JPEG]

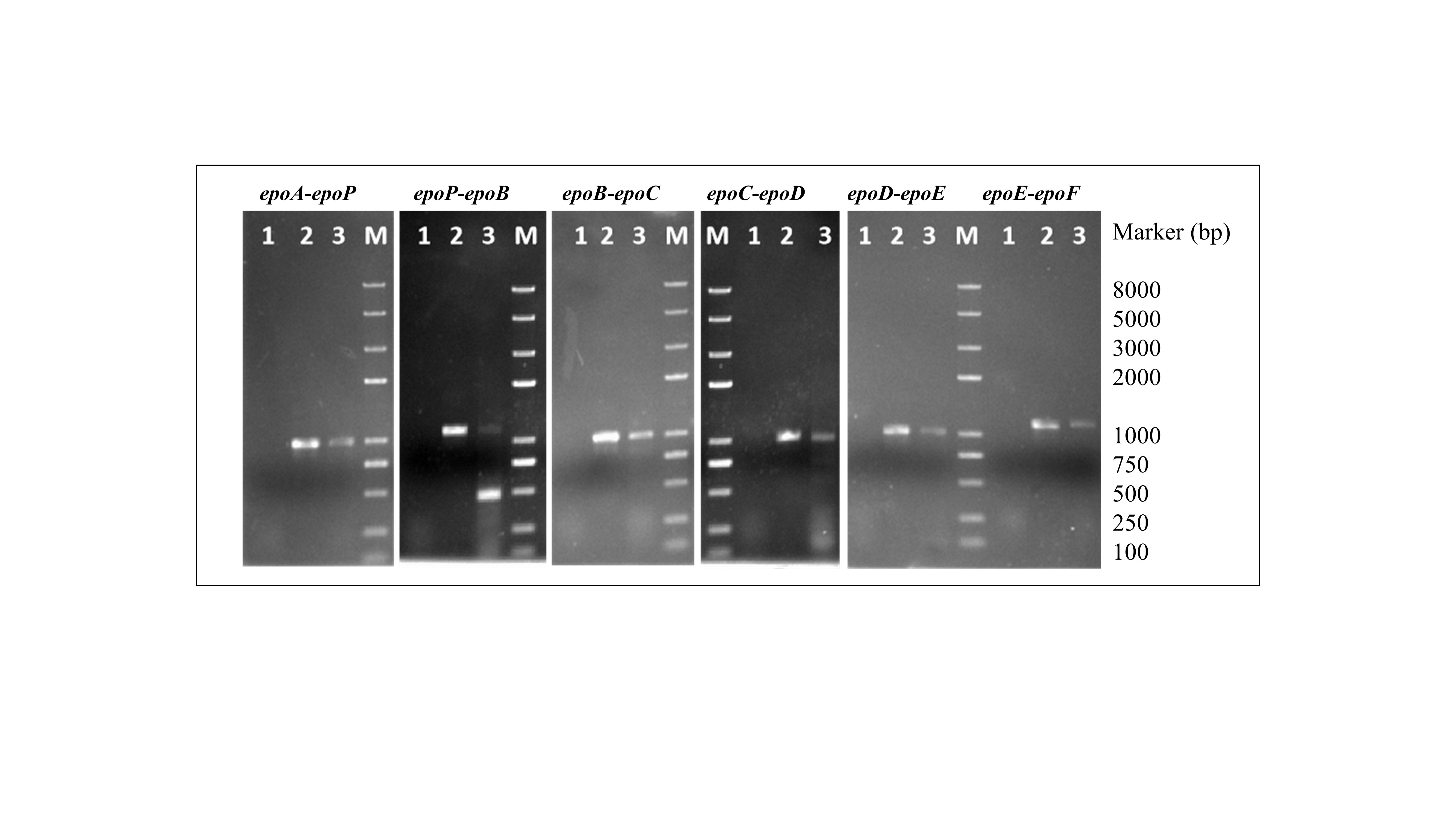

Supplement: Supplementary file 6 [file Image5.JPEG]

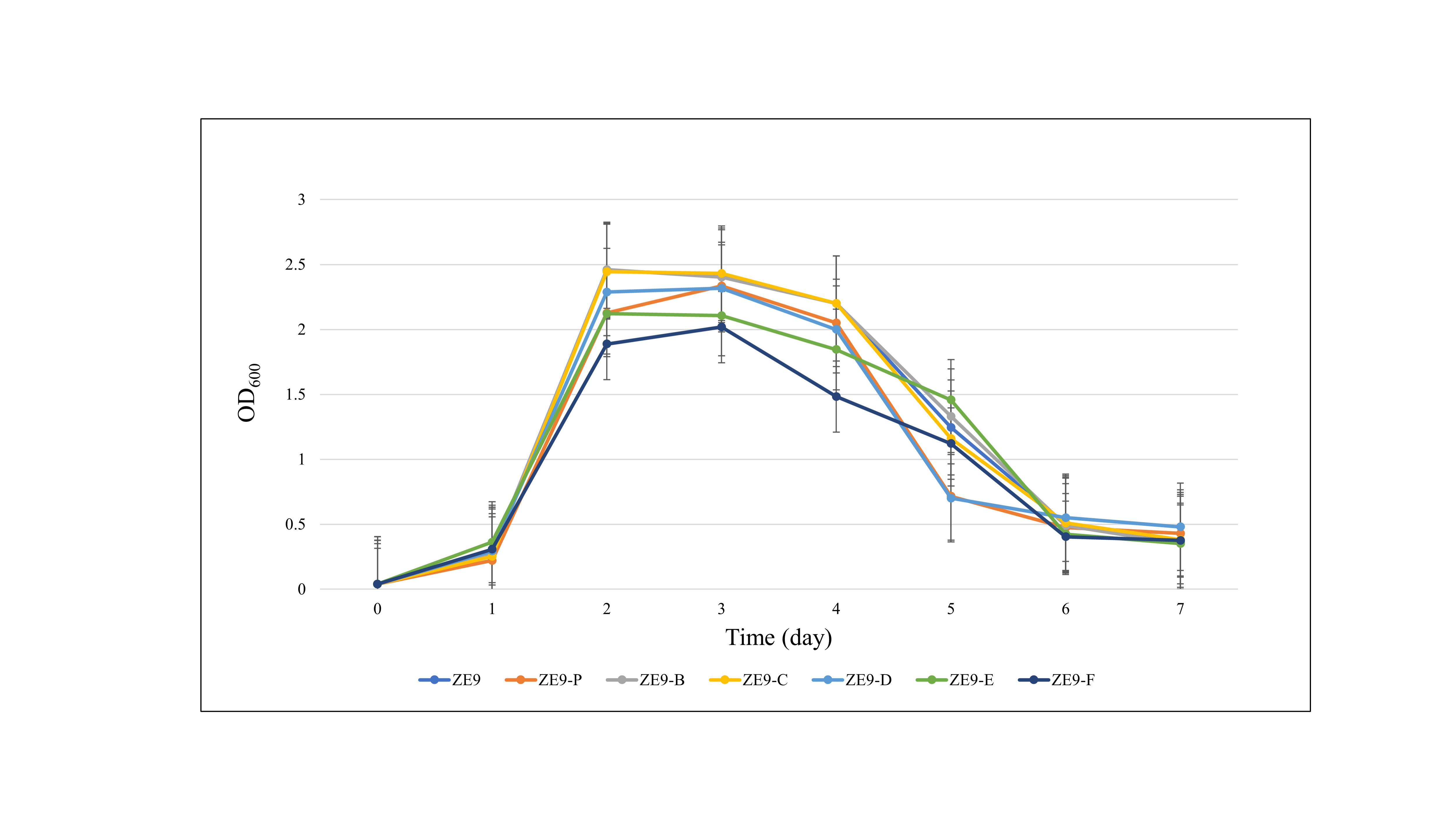

Supplement: Supplementary file 10 [file Image6.JPEG]
