# Supplementary material for: Internal Promoters and Their Effects on the Transcription of Operon Genes for Epothilone Production in Myxococcus xanthus
Source: Front Bioeng Biotechnol. 2021 Oct 27;9:758561. doi: 10.3389/fbioe.2021.758561 (PMC8579030; doi:10.3389/fbioe.2021.758561)
Supplement: Supplementary file 2 [file Table1.DOCX]

Table S1. Strains used in this study.

| Strains | Genotype or description | Source or references |
| --- | --- | --- |
| ***M. xanthus*** | | |
| DK1622 | Wild-type strains | D. Kaiser, University of Stanford |
| ZE9 | DZ2 with epothilone gene cluster | Zhu *et al*, 2014 |
| ZE9-CuOm | ZE9::pSWcuomxdCas9-Omega | Peng *et al*, 2018 |
| ZE9-P | ZE9-CuOm::pZJY41-sgRNA-P | This study |
| ZE9-B | ZE9-CuOm::pZJY41-sgRNA-B | This study |
| ZE9-C | ZE9-CuOm::pZJY41-sgRNA-C | This study |
| ZE9-D | ZE9-CuOm::pZJY41-sgRNA-D | This study |
| ZE9-E | ZE9-CuOm::pZJY41-sgRNA-E | This study |
| ZE9-F | ZE9-CuOm::pZJY41-sgRNA-F | This study |
| ZE9-AP | ZE9-CuOm::pZJY41-sgRNA-AP | This study |
| ZE9-AB | ZE9-CuOm::pZJY41-sgRNA-AB | This study |
| ZE9-APB | ZE9-CuOm::pZJY41-sgRNA-APB | This study |
| ZE9-DEF | ZE9-CuOm::pZJY41-sgRNA-DEF | This study |
| ZE5 | DZ2 with epothilone gene cluster | Zhu *et al*, 2014 |
| ZE5-DEF | ZE5::pSWcuomxdCas9-Omega::pZJY41-sgRNA-DEF | This study |
| ZE10 | DZ2 with epothilone gene cluster | Zhu *et al*, 2014 |
| ZE10-DEF | ZE10::pSWcuomxdCas9-Omega::pZJY41-sgRNA-DEF | This study |
| ***E. coli*** | | |
| DH5α |  |  |
| HB101 |  |  |
| pkk-232 | HB101::pkk-232-8 |  |
| pkk-aph | HB101::pkk-232-8-aphII | This study |
| pkk-P | HB101::pkk-232-8-P_epoP_ | This study |
| pkk-B | HB101::pkk-232-8-P_epoB_ | This study |
| pkk-C | HB101::pkk-232-8-P_epoC_ | This study |
| pkk-D | HB101::pkk-232-8-P_epoD_ | This study |
| pkk-E | HB101::pkk-232-8-P_epoE_ | This study |
| pkk-F | HB101::pkk-232-8-P_epoF_ | This study |
| pkk-CuOm | HB101::pSWcuomxdCas9-Omega | This study |
| pkk-Pa | pkk-CuOm::pkk-232-8-P_epoP_::pZJY41-sgRNA-P | This study |
| pkk-Ba | pkk-CuOm::pkk-232-8-P_epoB_::pZJY41-sgRNA-B | This study |
| pkk-Ca | pkk-CuOm::pkk-232-8-P_epoC_::pZJY41-sgRNA-C | This study |
| pkk-Da | pkk-CuOm::pkk-232-8-P_epoD_::pZJY41-sgRNA-D | This study |
| pkk-Ea | pkk-CuOm::pkk-232-8-P_epoE_::pZJY41-sgRNA-E | This study |
| pkk-Fa | pkk-CuOm::pkk-232-8-P_epoF_::pZJY41-sgRNA-F | This study |
