# Supplementary material for: Internal Promoters and Their Effects on the Transcription of Operon Genes for Epothilone Production in Myxococcus xanthus
Source: Front Bioeng Biotechnol. 2021 Oct 27;9:758561. doi: 10.3389/fbioe.2021.758561 (PMC8579030; doi:10.3389/fbioe.2021.758561)
Supplement: Supplementary file 7 [file Table2.DOCX]

**Table S2 Primers used in this study**

| Primer name | Primer sequence (5’-3’) | Restriction Site | Production |
| --- | --- | --- | --- |
| AP-F/R | CGTTCCGGAGCATGGCGCAG | | gene co-transcription: *epoA-epoP* |
|  | TCTCGCAACGATACGAGCCTC | |  |
| PB-F/R | ACGCGCGGCGCCCCTCCTG | | gene co-transcription: *epoP-epoB* |
|  | TTCAGCCTGTAGGAGACGTG | |  |
| BC-F/R | GTGAACCCGCGGCTGTGGG | | gene co-transcription: *epoB-epoC* |
|  | GGCAGCCGAGCGACCGTGC | |  |
| CD-F/R | GCAGTGGGTGGAGTTCTAC | | gene co-transcription: *epoC-epoD* |
|  | TCTTCGCGCGGCTGGTGCGC | |  |
| DE-F/R | GATTGGCGCTGTGGGCGGAG | | gene co-transcription: *epoD-epoE* |
|  | TACGCGTCCTGCTCCTCGCG | |  |
| EF-F/R | CGTCATGGGGACAGCCGTG | | gene co-transcription: *epoE-epoF* |
|  | TCTCGTCACACTCGGCCGG | |  |
| P_epoP_-F/R | cgcggatccTGCGGTATGTGAGCGATTC | BamHⅠ | pkk-232-P_epoP_ |
|  | cccaagcttCCGACCCAGCCAGTAGGA | HindⅢ |  |
| P_epoB_-F/R | cgcggatccGCTGAAGGTCTCCGAACACG | BamHⅠ | pkk-232-P_epoB_ |
|  | cccaagcttCCGATAGAGCCCTCGTAAGC | HindⅢ |  |
| P_epoC_-F/R | cgcggatccGCGGTTTCGTAAGGTGATG | BamHⅠ | pkk-232-P_epoC_ |
|  | cccaagcttCAATCTATCGGCTCGGTGA | HindⅢ |  |
| P_epoD_-F/R | cgcggatccTGACGGTAGCGAGGGCAGAC | BamHⅠ | pkk-232-P_epoD_ |
|  | cccaagcttCCTTCTCGGTCTGTCACGCAAT | HindⅢ |  |
| P_epoE_-F/R | cgcggatccGATGGCTTTCATACCCTGCTC | BamHⅠ | pkk-232-P_epoE_ |
|  | cccaagcttCTCCCTCGGTCCCTTACTCA | HindⅢ |  |
| P_epoF_-F/R | cgcggatccCTGCTTTCACGGTTCTGGC | BamHⅠ | pkk-232-P_epoF_ |
|  | cccaagcttAAGCGGAGCACCTCATCG | HindⅢ |  |
| sgRNA-R | CCCGCGGATGGGATTAGCACTACG | | pZJY41-sgRNA-X |
| SgRNA-A-F | CATGAAAATGGCCCTTTGAGGTTTTAGAGCTAGAAATAGC | | pZJY41-sgRNA-A |
| SgRNA-P-F | TCCGGGGGATGATGCTCGAGGTTTTAGAGCTAGAAATAGC | | pZJY41-sgRNA-P |
| SgRNA-B-F | CGGGACGCCTTGGAGGAGATGTTTTAGAGCTAGAAATAGC | | pZJY41-sgRNA-B |
| SgRNA-C-F | ACCGTACCGGCAACGCTGTTGGTTTTAGAGCTAGAAATAGC | | pZJY41-sgRNA-C |
| SgRNA-D-F | TGCGGCCGGTATCCTGGACGAGTTTTAGAGCTAGAAATAGC | | pZJY41-sgRNA-D |
| SgRNA-E-F | TGGATGTATCCCAAGGTGCTGTTTTAGAGCTAGAAATAGC | | pZJY41-sgRNA-E |
| SgRNA-F-F | AGCTCTTCTTCCGAAATGCCGGTTTTAGAGCTAGAAATAGC | | pZJY41-sgRNA-F |
| BK-F | cgggatccCTCGTCGCGGCGATGAAGCA | BamHⅠ | pZJY41-sgRNA-AP; pZJY41-sgRNA-AB;  pZJY41-sgRNA-APB; pZJY41-sgRNA-DEF |
| BK-R | ggggtaccAAAAAAAGCACCGACTCGGTGCCAC | KpnⅠ |  |
| EcoR-F | cggaattgCTCGTCGCGGCGATGAAGCA | EcoRⅠ |  |
| EcoR-R | cggaattgAAAAAAAGCACCGACTCGGTGCCAC | EcoRⅠ |  |
| Nde-F | cccatatgCTCGTCGCGGCGATGAAGCA | NdeⅠ |  |
| Nde-R | cccatatgAAAAAAAGCACCGACTCGGTGCCAC | NdeⅠ |  |
| QgapA-F/R | GCCCTGGAAGAGCCTGAACG | | *gapA* gene (RT-qPCR) |
|  | TGGAGACGATGTGGTGCTTGG | |  |
| QA-F/R | GCGTTCCACTCACCGCTCAT | | *epoA* gene (RT-qPCR) |
|  | GCCTTCCCGCTCAGATTGCT | |  |
| QP-F/R | GCTCAACATAACGCTCTTCAACC | | *epoP* gene (RT-qPCR) |
|  | CTGGACCTCGATACCGCTCA | |  |
| QB-F/R | ATGGAAGAACAAGATTCCTC | | e*poB* gene (RT-qPCR) |
|  | CTCGGAGAAGCGCTGCACGG | |  |
| QC-F/R | GAAGATGCGGTGAGGTTGGTGG | | *epoC* gene (RT-qPCR) |
|  | TCGGACGCTGCGATGGCTAC | |  |
| QD-F/R | GTGACAGACCGAGAAGGCC | | *epoD* gene (RT-qPCR) |
|  | CCACGATGGCGATCGGCTCG | |  |
| QE-F/R | GCACCGTTTGCGTTAGTAGGG | | *epoE* gene (RT-qPCR) |
|  | GCTTGGCTATTATGTCGGTCTCC | |  |
| QF-F/R | GGAGCAAGCGAATCAGAGTG | | *epoF* gene (RT-qPCR) |
|  | CGTGGTATCGGGTGAGGAC | |  |
