# Supplementary material for: Internal Promoters and Their Effects on the Transcription of Operon Genes for Epothilone Production in Myxococcus xanthus
Source: Front Bioeng Biotechnol. 2021 Oct 27;9:758561. doi: 10.3389/fbioe.2021.758561 (PMC8579030; doi:10.3389/fbioe.2021.758561)
Supplement: Supplementary file 8 [file Table3.DOCX]

Table S3 Plasmids used in this study.

| Plasmids | Genotype or description | Source or references |
| --- | --- | --- |
| pSWU30 | Site-specific integration vector with Mx8 attP integration site, Tet^r^ | Mignot Tâm, CNRS(Centre national de la recherché scientifique) |
| pSWcuomxdCas9-Omega | codon-optimized dcas9 gene, with copper-inducible promoter P_cuoA_ sequence, inserted into EcoRⅠ/HindⅢ sites of pSWU30, Tet^r^ | Peng *et al*,2018 |
| pKK-232-8 | Amp^r,^ |  |
| pKK-232-aphII | *aphII* promoter sequence inserted into BamHⅠ/HindⅢ sites of pKK232-8, Cm^r,^, Amp^r,^ | This study |
| pKK-232-P_epoP_ | *P_epoP_* inserted into BamHⅠ/HindⅢ sites of pKK232-8, Cm^r,^, Amp^r,^ | This study |
| pKK-232-P_epoB_ | *P_epoB_* inserted into BamHⅠ/HindⅢ sites of pKK232-8, Cm^r,^, Amp^r,^ | This study |
| pKK-232-P_epoC_ | *P_epoC_* inserted into BamHⅠ/HindⅢ sites of pKK232-8, Cm^r,^, Amp^r,^ | This study |
| pKK-232-P_epoD_ | *P_epoD_* inserted into BamHⅠ/HindⅢ sites of pKK232-8, Cm^r,^, Amp^r,^ | This study |
| pKK-232-P_epoE_ | *P_epoE_* inserted into BamHⅠ/HindⅢ sites of pKK232-8, Cm^r,^, Amp^r,^ | This study |
| pKK-232-P_epoF_ | *P_epoF_* inserted into BamHⅠ/HindⅢ sites of pKK232-8, Cm^r,^, Amp^r,^ | This study |
| pZJY41 | Km^r^, Amp^r^ | Zhao *et al.* 2008 |
| pZJY41-sgRNA-P | sgRNA scaffold with spacerP inserted into BamHⅠ/KpnⅠ sites of pZJY41, Km^r^ | This study |
| pZJY41-sgRNA-B | sgRNA scaffold with spacerB inserted into BamHⅠ/KpnⅠ sites of pZJY41, Km^r^ | This study |
| pZJY41-sgRNA-C | sgRNA scaffold with spacerC inserted into BamHⅠ/KpnⅠ sites of pZJY41, Km^r^ | This study |
| pZJY41-sgRNA-D | sgRNA scaffold with spacerD inserted into BamHⅠ/KpnⅠ sites of pZJY41, Km^r^ | This study |
| pZJY41-sgRNA-E | sgRNA scaffold with spacerE inserted into BamHⅠ/KpnⅠ sites of pZJY41, Km^r^ | This study |
| pZJY41-sgRNA-F | sgRNA scaffold with spacerF inserted into BamHⅠ/KpnⅠ sites of pZJY41, Km^r^ | This study |
| pZJY41-sgRNA-AP | sgRNA scaffold with spacerA inserted into NdeⅠ sites of pZJY41, sgRNA scaffold with spacerP inserted into BamHⅠ/KpnⅠ sites of pZJY41, Km^r^ | This study |
| pZJY41-sgRNA-AB | sgRNA scaffold with spacerA inserted into NdeⅠ sites of pZJY41, sgRNA scaffold with spacerB inserted into BamHⅠ/KpnⅠ sites of pZJY41, Km^r^ | This study |
| pZJY41-sgRNA-APB | sgRNA scaffold with spacerA inserted into NdeⅠ sites of pZJY41, sgRNA scaffold with spacerP inserted into BamHⅠ/KpnⅠ sites of pZJY41, sgRNA scaffold with spacerB inserted into EcoRI sites of pZJY41, Km^r^ | This study |
| pZJY41-sgRNA-DEF | sgRNA scaffold with spacerD inserted into NdeⅠ sites of pZJY41, sgRNA scaffold with spacerE inserted into BamHⅠ/KpnⅠ sites of pZJY41, sgRNA scaffold with spacerF inserted into EcoRI sites of pZJY41, Km^r^ | This study |
