# Supplementary material for: Internal Promoters and Their Effects on the Transcription of Operon Genes for Epothilone Production in Myxococcus xanthus
Source: Front Bioeng Biotechnol. 2021 Oct 27;9:758561. doi: 10.3389/fbioe.2021.758561 (PMC8579030; doi:10.3389/fbioe.2021.758561)
Supplement: Supplementary file 9 [file Table4.DOCX]

**Table S4 Prediction of the promoters of the epothilone gene cluster from So0157-2.**

| Neural Network Promoter Prediction | | | Softberry BPROM | | | |
| --- | --- | --- | --- | --- | --- | --- |
| Gene | Sequence of promoter | **Distance (bp)** | Gene | **-10 box** | **-35 box** | **Distance (bp)** |
| *epoP* | CAGCTTCTGAACGAGCTCGAGCACCAGGGTATCAAGCTGGCGGCCGATGG | +12 | ***epoP*** | ATCTATGAC | TTGCGA | +492 |
| *epoB* | CTCGTGCTGACGCAGCCATGGCTGGATGGTAAACTGTCATGGCCGCCGGG | -2513 | ***epoB*** | TGGTAAACT | CTGACG | -2507 |
|  | GACTTCTTGAGGACCAAACTACCCGAGTATATGGTGCCTACAGTCTTCGT | -596 |  | GTATATGGT | TTGAGG | -590 |
|  | | |  | GCGTAATCT | ATGTCG | +39 |
|  |  |  |  | AGCGATGAT | TTGACG | +387 |
| *epoC* | ACATGACTACTCGCGGTCCTACGGCACAGCAGAATCCGCTGAAACAAGCG | -2 | ***epoC*** | AGCTAAATT | CTGACG | -38 |
| *epoD* | None | | ***epoD*** | CGCTCTCCT | TTGACA | -841 |
| *epoE* | GATTGATTGAGGGGTGGCAGAAGTACGAAGATGATCTTCGTACCGACCAT | -2473 | ***epoE*** | GAGTACCAT | TTGCTG | -2774 |
|  | TACGCGTTGGAATGGCAGCGCAAAGACCCTATACCAGAGGCTCCGGCAGC | -1964 |  | TACGAAGAT | TTGATT | -2471 |
|  | TTCTTGGACGCCATCGCGCATCACCGGTGTAGGCTGGGCCTTACAGCGAT | -773 |  | GTGTAGGCT | TGGACG | -769 |
| *epoF* | GTCTTGTGGATGACGGGCTGCTGATGCAGCAGACTCCGGCGCGGTTCCGC | -1733 |  | CGTGATAAT | ATGCTC | +678 |
|  | GCCTTCCTGGAAACTCGCAGCATAGGAGATTTTATGACGCAGGAGCAAGC | -33 | ***epoF*** | GGGAATGAT | TTTCCT | -2303 |
|  | GTTTGGATACCACCCCGCGTTCCGGAACATCGAATCACTCAACGTCATCT | +1187 |  | ATCTACAAT | GTGCCT | -303 |

Distance means the number of bases between the start base of the predicted internal promoter and the translation initiation codon of the downstream gene. “+” means downstream of the transcription start site, “-” means upstream of the transcription start site.
